# Supplementary material for: Irreversible furin cleavage site exposure renders immature tick-borne flaviviruses fully infectious
Source: Nat Commun. 2025 Aug 12;16:7491. doi: 10.1038/s41467-025-62750-6 (PMC12343913; doi:10.1038/s41467-025-62750-6)
Supplement: Supplementary file 1 — Supplementary Information [file 41467_2025_62750_MOESM1_ESM.pdf]

Supplementary information

Irreversible furin cleavage site exposure renders immature tick-borne flaviviruses fully infectious

Jiří Holoubek[1,2,3], Jiří Salát[1,2,3], Milos Matkovic[4], Petr Bednář[1,2,3], Pavel Novotný[5,6], Martin Hradilek[5], Taťána Majerová[5], Ebba Rosendal[7], Luděk Eyer[1,2,3], Andrea Fořtová[1,2,3], Michaela Beránková[1,2,3], Lesley Bell-Sakyi[8], Anna K. Överby[7], Andrea Cavalli[4,9], Massimiliano Bonomi[10], Félix A. Rey[11*] and Daniel Růžek[1,2,3*]

[1]Department of Experimental Biology, Faculty of Science, Masaryk University, CZ-62500, Brno, Czech Republic

[2]Laboratory of Emerging Viral Diseases, Veterinary Research Institute, CZ-62100, Brno, Czech Republic

[3]Institute of Parasitology, Biology Centre of the Czech Academy of Sciences, CZ-37005, Ceske Budejovice, Czech Republic

[4]Institute for Research in Biomedicine, Università della Svizzera Italiana, Bellinzona, Switzerland

[5]Institute of Organic Chemistry and Biochemistry of the Czech Academy of Sciences, CZ-16610, Prague, Czech Republic

[6]Department of Physical and Macromolecular Chemistry, Faculty of Science, Charles University, CZ-12843, Prague, Czech Republic

[7]Department of Clinical Microbiology, Laboratory for Molecular Infection Medicine Sweden (MIMS), Umeå University, SE-90187, Umeå, Sweden

[8]Department of Infection Biology and Microbiomes, Institute of Infection, Ecological and Veterinary Sciences, University of Liverpool, Liverpool L3 5RF, United Kingdom

[9] Swiss Institute of Bioinformatics, Lausanne, Switzerland

[10]Institut Pasteur, Université Paris Cité, CNRS UMR 3528, Computational Structural Biology Unit, Paris, France

[11]Institut Pasteur, Université de Paris Cité, CNRS UMR 3569, Structural Virology Unit, 75015 Paris, France.

*Authors for Correspondence: Daniel Ruzek, ruzekd@paru.cas.cz; Félix A. Rey, felix.rey@pasteur.fr

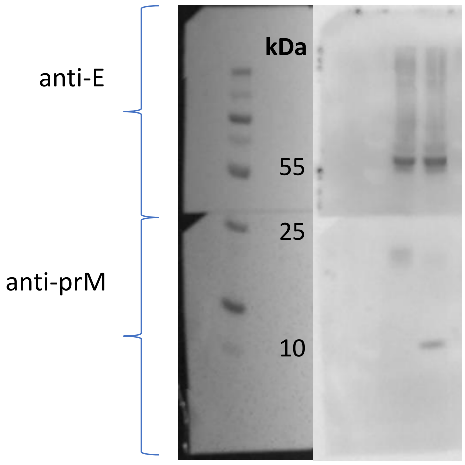

Supplementary Fig. 1 | Unmodified western blot image corresponding to Fig. 1b. Western blot membrane was divided into two parts to enable separate incubation with different primary and secondary antibodies. Image was acquired with a GE Amersham imager 680.

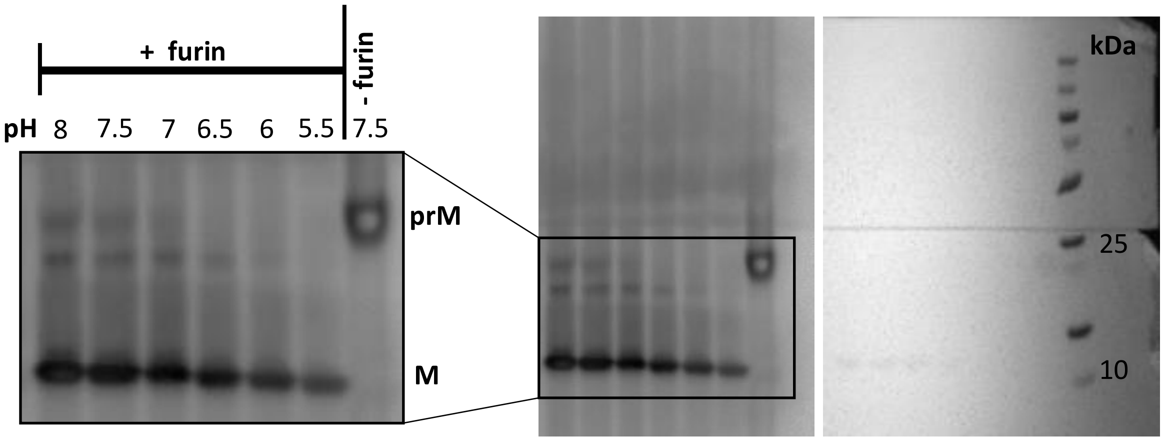

Supplementary Fig. 2 | pH dependence of furin cleavage of prM-TBEV. Western blot analysis showed efficient furin cleavage over the entire pH range from 5.5 to 8.

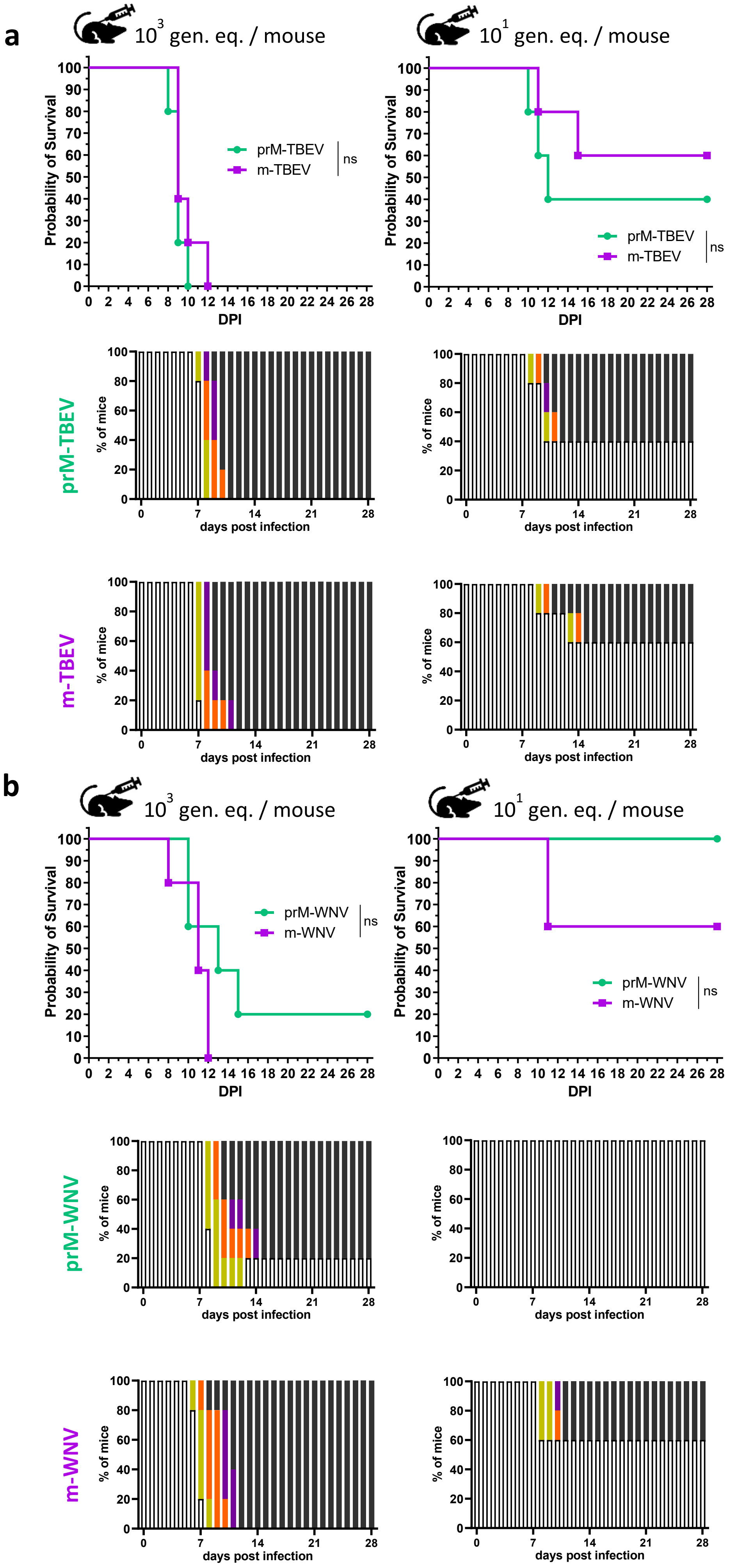
a
10³ gen. eq. / mouse
Probability of Survival
DPI
prM-TBEV
m-TBEV
ns
10¹ gen. eq. / mouse
prM-TBEV
m-TBEV
% of mice
days post infection
b
10³ gen. eq. / mouse
10¹ gen. eq. / mouse
prM-WNV
m-WNV
ns
Probability of Survival
DPI
% of mice
days post infection

Supplementary Fig. 3 | Comparison of the pathogenicity of prM-TBEV/m-TBEV and prM-WNV/m-WNV in a mouse model. a, Four groups of adult BALB/c mice (n = 5, 5) were subcutaneously infected with two different doses (10^1 and 10^3 genome equivalents per mouse) of prM-TBEV (two groups) or m-TBEV (two groups), respectively. In the case of prM-TBEV, the infectious dose calculation corresponds to ~ 4 and 400 PFU for 10^1 and 10^3 gen. eq. per mouse, respectively. Survival rates and clinical scores were monitored for 28 days. Clinical score was evaluated as follows: 1, no signs; 2, piloerection; 3, hunched back; 4, paralysis; and 5, death **b,** Similar to the above experiment, four groups of mice were infected with either prM-WNV or m-WNV, and survival was monitored for 28 days. In the case of prM-WNV, the infectious dose calculation corresponds to ~ 0.07 and 7 PFU for 10^1 and 10^3 gen. eq. per mouse, respectively. Clinical signs of disease were assessed as described above. Survival rates were statistically evaluated using the log-rank Mantel-Cox test.

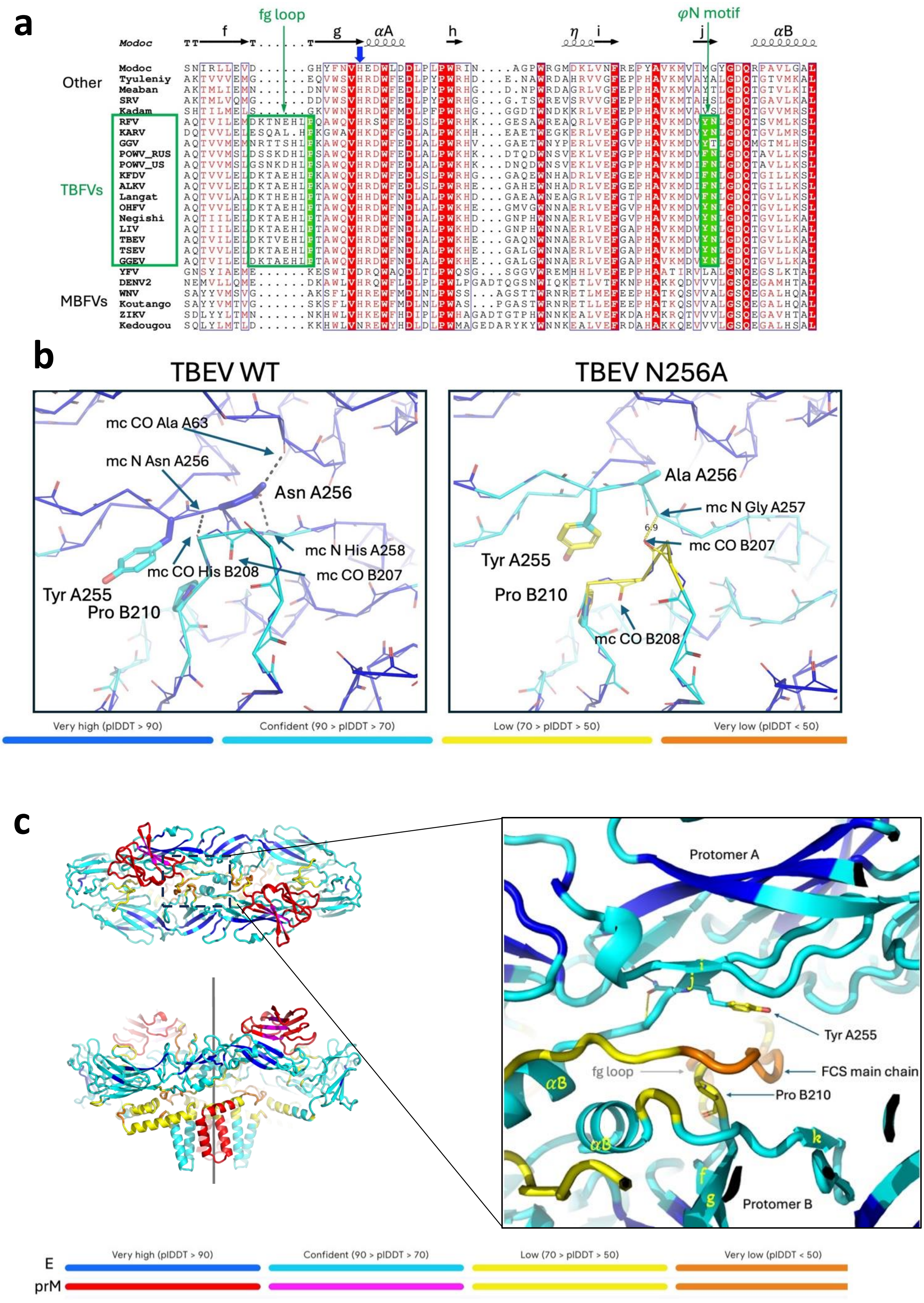
a
fg loop
φN motif
Modoc
f
g
αA
h
η
i
j
αB
Other
Modoc
Tyuleniy
Meaban
SRV
Kadam
TBFVs
RFV
KARV
GGV
POWV_RUS
POWV_US
KFDV
ALKV
Langat
OHFV
Negishi
LIV
TBEV
TSEV
GGEV
MBFVs
YFV
DENV2
WNV
Koutango
ZIKV
Kedougou
b
TBEV WT
TBEV N256A
mc CO Ala A63
mc N Asn A256
Asn A256
mc N His A258
mc CO His B208
mc CO B207
Tyr A255
Pro B210
Ala A256
mc N Gly A257
6.9
mc CO B207
Tyr A255
Pro B210
mc CO B208
Very high (pIDDT > 90)
Confident (90 > pIDDT > 70)
Low (70 > pIDDT > 50)
Very low (pIDDT < 50)
c
Protomer A
Tyr A255
fg loop
FCS main chain
Pro B210
αB
αB
Protomer B
E
prM
Very high (pIDDT > 90)
Confident (90 > pIDDT > 70)
Low (70 > pIDDT > 50)
Very low (pIDDT < 50)

Supplementary Fig. 4 | (prM/E)$_2$ dimer interface. a, Amino acid sequence alignment of E iform flaviviruses that infect vertebrates, highlighting in green two key TBFV-specific motifs identified here: the conserved proline in the fg loop insertion and the "φN" where stands for tyrosine or phenylalanine. Note that the conserved asparagine in this motif is replaced by threonine in one instance, whose side chain could also make a hydrogen bond with the main chain. A vertical blue arrow points to position 216 (TBEV numbering) in the $\alpha\mathrm{A}$-$\alpha\mathrm{B}$ groove, believed to play a role in unzippering via repulsion with prM His95. The alignment shows that His216 is not conserved across all flaviviruses, indicating that other residues may contribute to unzippering for those viruses. **b,** AlphaFold3 prediction of a wild type sE dimer (left) and a N256A mutant sE dimer (right). The two chains of the sE dimer are labeled A and B and are displayed as Cα trace superposed with main chain atoms as lines color coded by atoms, with oxygen red, nitrogen blue, and carbon atoms according to AlphaFold confidence in the prediction (plDDT score, as indicated at the bottom by the colored bars). Dotted lines indicate hydrogen bonds. Note that the prediction for the wt sE dimer recapitulates the inter-chain interactions observed experimentally, but not the N256A mutant: in the absence of the hydrogen bond made by the Asn256 side chain, the main chain nitrogen at this position is not in the right geometry for the inter-chain hydrogen bond, and the fg loop re-organizes in a different conformation, and the CH/ interaction between Tyr255 and Pro210 broken. mc CO= main chain carbonyl. Mc N=main chain amine. **c,** AlphaFold3 prediction of a wild type sE dimer The plDDT color code is represented differently for prM and E, to distinguish the two in the left panel. A vertical bar in the bottom-left panel represents the molecular 2-fold axis of the (prM/E)$_2$ dimer. The inset (right panel) shows that the AF3 prediction places the main chain of the linker in between the side chains of Tyr255 and Pro210 of the two E protomers facing each other63thn . The relevant β-strands and α-helices in protein E are labeled in yellow.

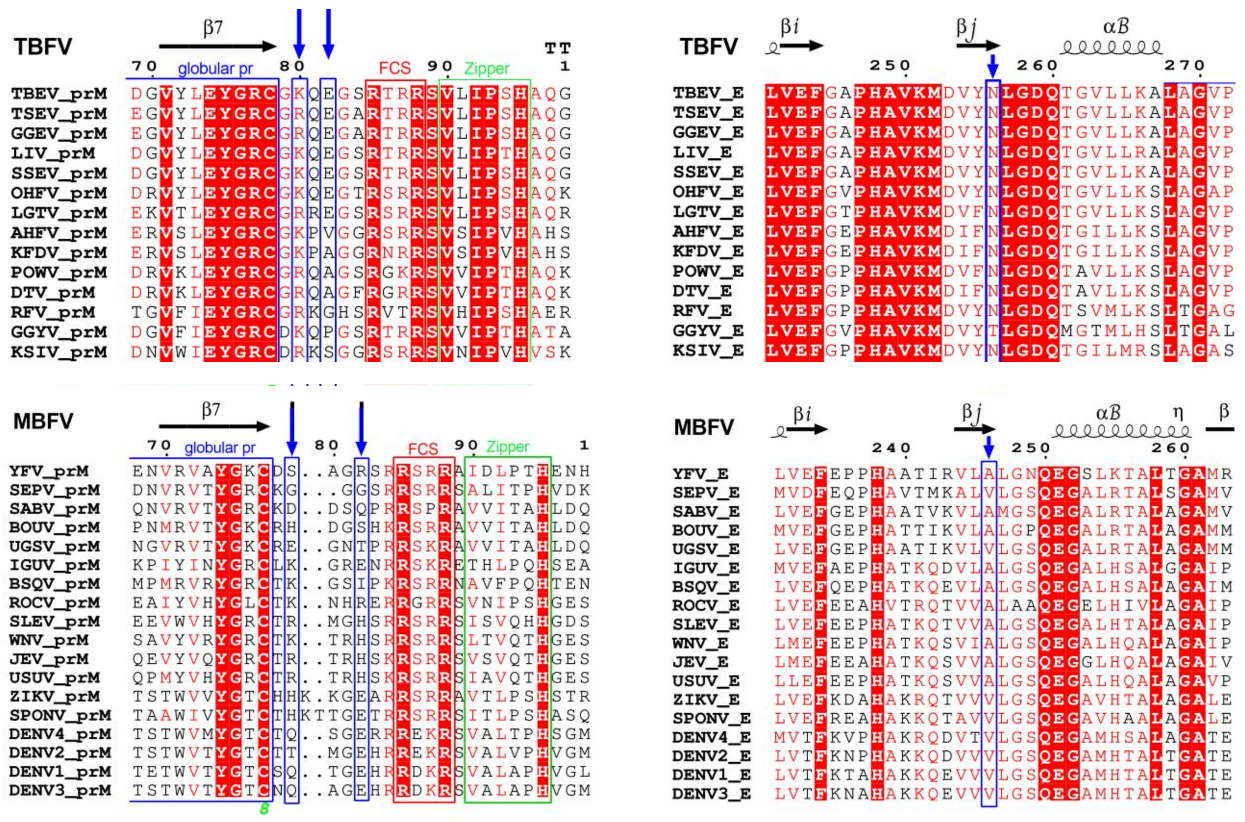

Supplementary Fig. 5 | Sequence alignment of flavivirus prM and E proteins showing the regions where mutations were introduced in this study (solid blue arrows). On the left, in TBFVs, the residue at prM position 80 is consistently basic, whereas in MBFVs, this position is highly variable. In contrast, position 82 never contains a basic residue and often features a negatively charged amino acid. Upstream of this region is the globular pr domain, while downstream are the furin cleavage site (FCS, framed in red) and the zipper region (framed in green). On the right, the alignment of E proteins highlights the mutation at position 256 (TBEV numbering) within β-strand j (solid blue arrow), and also includes the αB helix region.

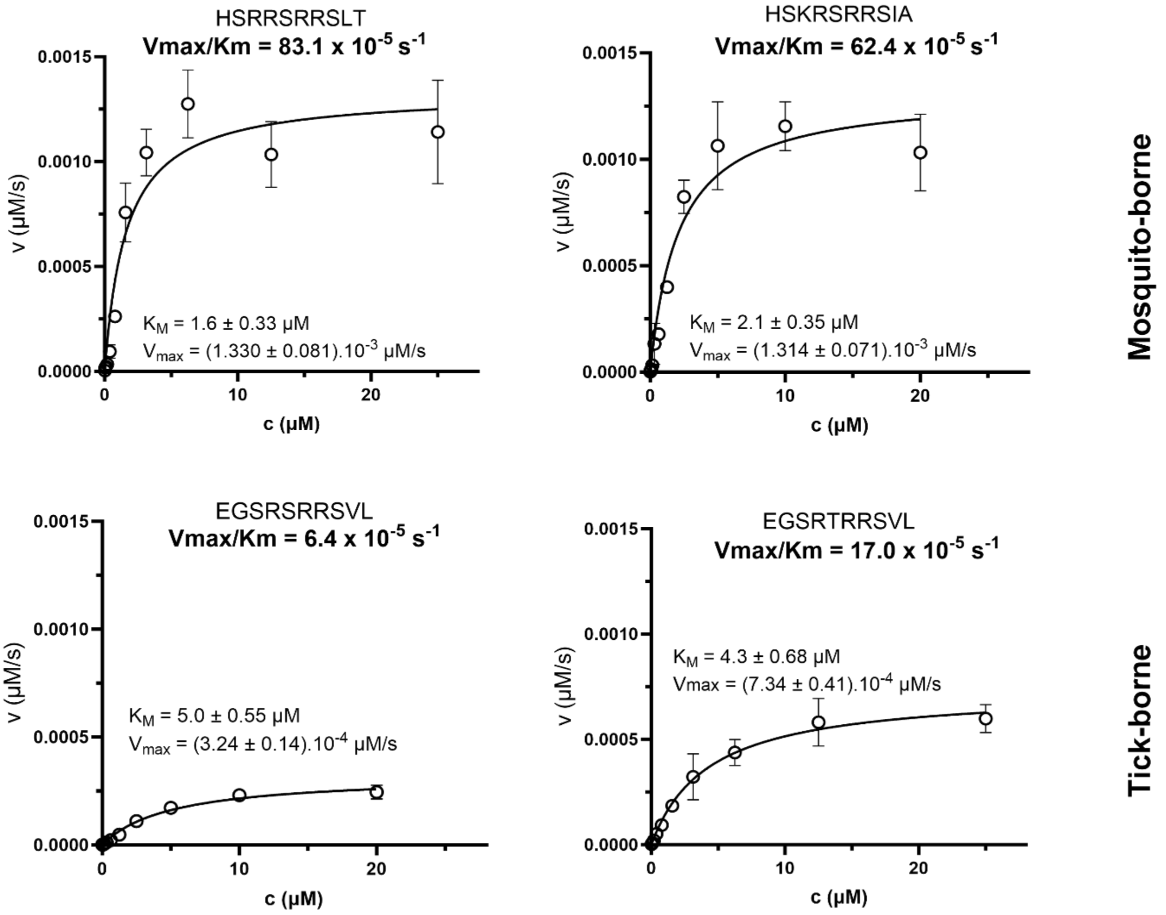

Supplementary Fig. 6 | Determination of Km/Vmax values. The substrate-dose-dependent initial velocity of cleavage was measured at pH 7.0 in buffer (25 mM acetic acid, 25 mM MES, 25 mM glycine, 1 mM $CaCl_2$) for each peptide substrate, in triplicate. Reactions were started by addition of furin (final concentration of 10 ng mL^{-1}). Km and Vmax values were fitted to the Michaelis-Menten equation using GraphpadPrism software. The resultant Vmax/Km indicates that the studied mosquito-borne prM-derived peptides were more efficiently cleaved by furin, compared to substrates derived from tick-borne flaviviruses.

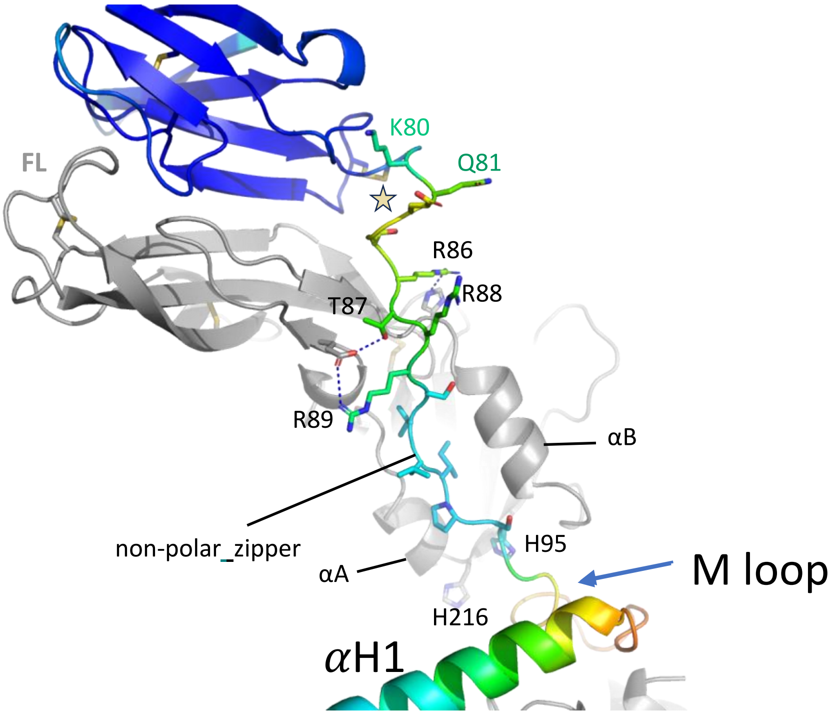

Supplementary Fig. 7 | The prM/E interfaces in TBEV. Detailed view of the TBEV prM/E protein complex in the zipper region, highlighting interactions along the cleavage site. The E protein is shown in grey, and the prM structure is coloured according to AlphaFold prediction accuracy (blue – high confidence, red – low confidence). The star marks the last disulfide bond at the end of the globular part of prM. The AF3 predictions are shown color-coded by plDDT (>90 blue, < 90 cyan >70, < 70 yellow > 50, <50 orange).

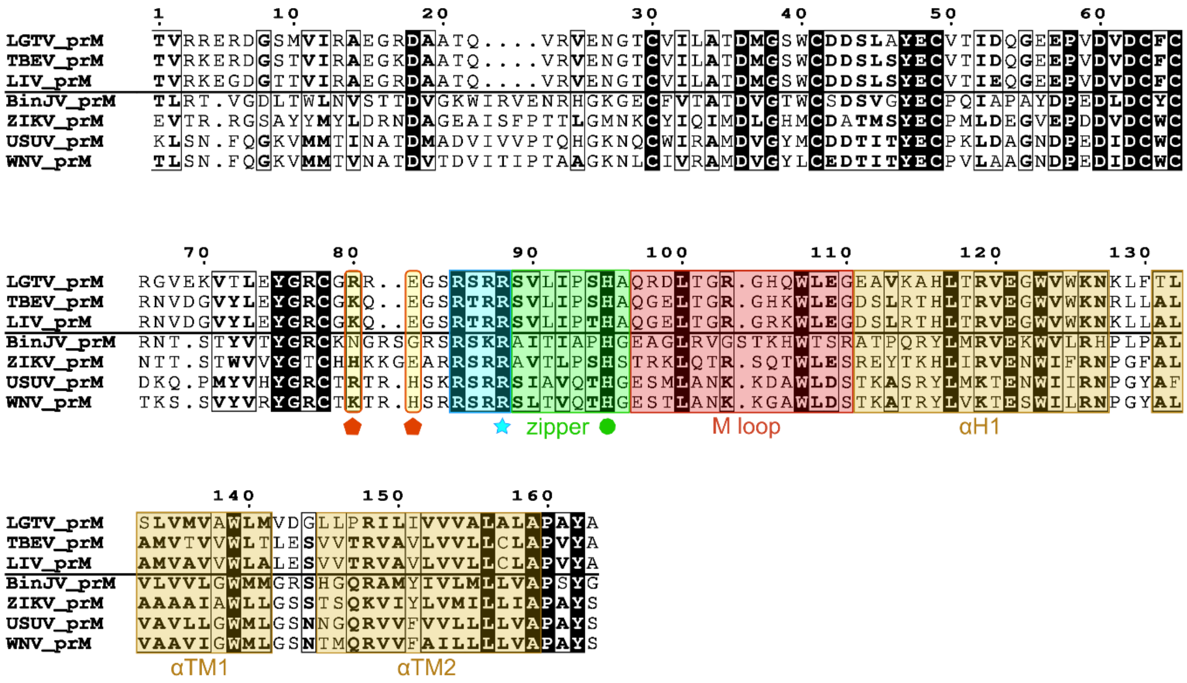

Supplementary Fig. 8 | Multiple sequence alignment of full-length prM proteins from flaviviruses analyzed in this study, with Binjari virus included for comparative context. The alignment highlights furin cleavage site (blue), zipper region (green) with highly conserved histidine H95 (green circle) and amino acid residues K80 and E82 (orange), which were mutated in rTBEV. The downstream region, consisting of the M loop and the C-terminal helical regions (αH1, αTM1 and αTM2), are shown in red and ochre, respectively. Percentage of equivalent residues was calculated considering the physico-chemical properties. Fully conserved residues are shown on a black background, and residues in black frames indicate that over 70% of residues share similar properties. Alignment was performed using ClustalW, with subsequent graphical rendering using ESPript 3.0.

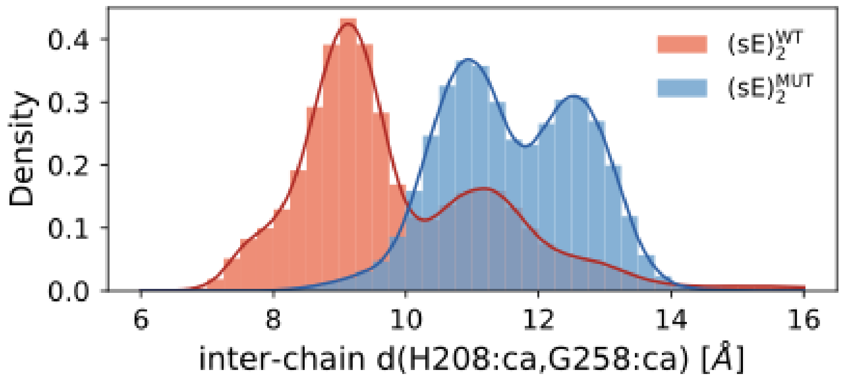

Supplementary Fig. 9 | Molecular Dynamics simulations of $(sE)_2$ dimers. Distributions of distances between the Cα atoms of residues H208 and G258. Raw normalized histograms are represented by bars, kernel density estimations as solid lines; the red and blue colours indicate $(sE)_2^{WT}$ and $(sE)_2^{MUT}$, respectively.

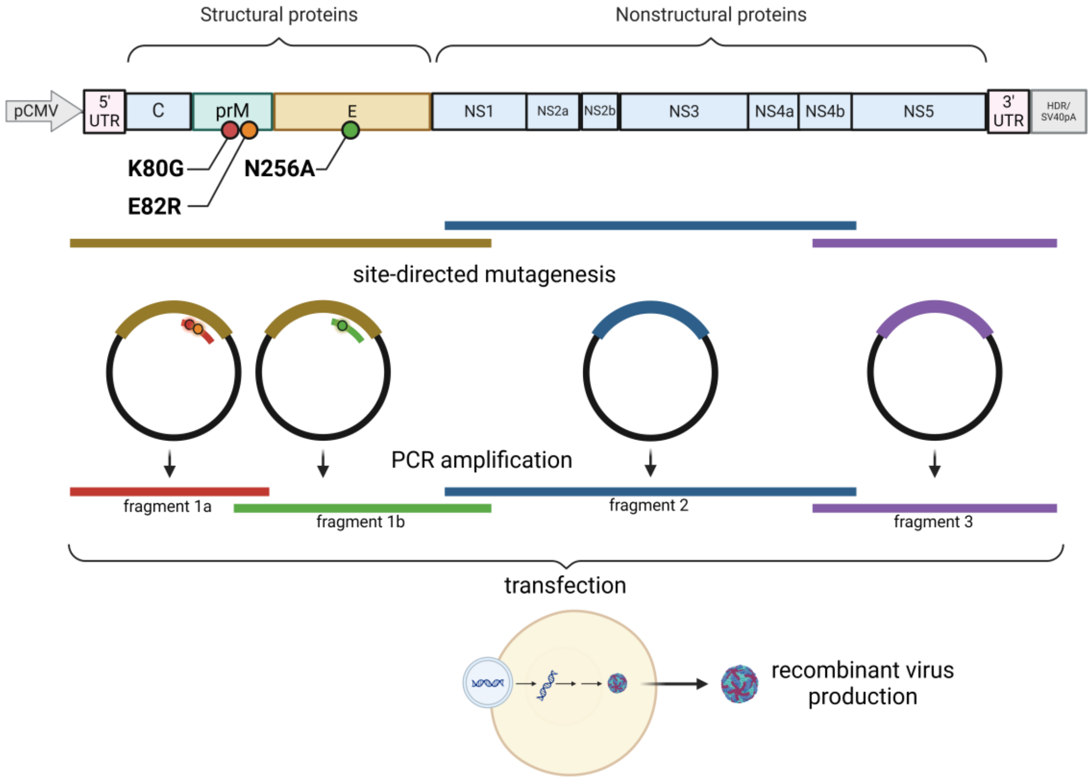

Supplementary Fig. 10 | Scheme representing the site-directed mutagenesis approach using infectious subgenomic amplicons. Mutated sites in the prM (K80G, E82R) and E (N256A) proteins were introduced using modified primers, and are highlighted. Overall, four amplified DNA fragments with overlapping ends were transfected into BHK-21 cells, followed by harvesting of the recombinant virus. Created in BioRender. Ruzek, D. (2025) https://BioRender.com/4n4b27g.

Supplementary Table 1 | Primers used to generate overlapping amplicons of TBEV fragments to produce recombinant virus rTBEV. The corresponding sequencing results with sequencing primers are shown below.

Primer ID	Sequence (5' - 3')
**mut_prM_site_1+2_FWa**	GGGGGTCAGCGTGGCTCACGGACAAGGCGC
**mut_prM_site_1+2FWb**	GGGGGTCAGCGTGGCTCACGGACAAGG
**mut_prM_site_1+2_R**	GCCACGCTGACCCCCACAGCGTCCATACTCC
**mut_Env_site_3_FW**	TGTACGCACTCGGAGACCAGACTGG
**mut_Env_site_3_R**	TCTCCGAGTGCGTACACATCCATC
**TBEV_1A_Fseq**	TGTCTACGCTTCGCGTTGCACACA
**TBEV_2B_Rseq**	TTCGTTCCGTGTCCACAGCGCA
**TBEV_2C_Fseq**	GGTCATTACTCTGTTGGGGA
**TBEV_2D_Rseq**	ACACATCCATTGAAGGCTTC

>1A-E_1A-primer_EF70119093

GATTTGTGACTGGTACTCAGGGGGACTACGAGGGTCACCTTGGTGCTGGAACTGGGTGGATGTGTTACCATAA
CAGCTGAGGGGAAGCCTTCAATGGATGTGTGGCTTGACGCCATTTACCAGGAGAACCCTGCTCAGACACGTGA
GTACTGTTTACACGCCAAGTTGTCGGACACTAAGGTTGCAGCCAGATGCCCAACAATGGGACCAGCCACTTTG
GCTGAAGAACACCAGGGTGGTACAGTGTGCAAGAGAGATCAGAGTGATCGAGGCTGGGGCAACCACTGTGG
ACTTTTTGGAAAGGGTAGCATTGTGGCCTGTGTCAAGGTGGCTTGTGAGGCAAAAAAGAAAGCCACAGGACA
TGTGTACGACGCCAACAAAATAGTGTACACGGTCAAAGTCGAACCACACACGGGAGACTATGTTGCCGCAAAC
GAGACACATAGTGGGAGGAAGACGGCATCCTTCACAGTTTCTTCAGAGAAAACCATTCTGACTATGGGTGAGT
ATGGAGATGTGTCTCTGTTGTGTAGGGTCGCTAGTGGCGTTGACTTGGCCCAGACCGTCATCCTTGAGCTTGA
CAAGACAGTGGAACACCTTCCAACGGCTTGGCAGGTCCACAGGGACTGGTTTAATGATCTGGCTCTGCCATGG
AAACATGAGGGAGCGCGAAACTGGAATAACGCAGAAAGATTGGTTGAATTTGGGGCTCCTCATGCTGTCAAG
ATGGATGTGTACGCACTCGGAGACCAGACTGGAGTGTTACTGAAGGCTCTCGCTGGGGTTCCTGTGGCACACA
TTGAGGGAACCAAGTACCACCTGAAGAGTGGCCATGTGACCTGCGAAGTGGGACTGGAAAAACTGAAGATGA
AAGGTCTTACGTACACAATGTGTGACAAAACAAAGTTCACATGGAAGAGAGCTCCAACAGACAGTGGGCATG
ATACAGTGGTCATGGAAGTCACATTCTCTGGAACAAAGCCCTGTAGGATCCCAGTCAGGGCAGTGGCACATGG
ATCTCCAGATGTGAACGTGGCCATGCTGATAACGCCAAACCCAACATTGAAAACAATGGAGGTGGCTTCATAG
AGATGCAGCTGCCCCCAGGAGACACATCATCTATGTTGGGGAACTGAATTATC

>1A-E_2B-primer_EF70119094

CACTCCAAGGGGTCATGGGCCAAAACCAGAACTCCAGCCAAGAGAAAGCTCATGGACATTGTAGGGTTTCTCA
TGTTCAGGCCCAACCAAGCCAATGCCACTCCTAATAGAAGCTTTGGTAGAAACCCCACTCCCCCGAAGATGCTG
TTGAAAGCACCACCAAGGACCGTGTGCAACGCCTTCCCAATTGAACTCAGAAAGCCTCCAGCAGAACCGAAGT

CCCAGGCGTGCTCCCCTATCACTGTCAGTCTTTCTATGCCTTTCTTGGTCTTTTGGAAAACTCTTCCGATGCTACT
CCCTTTTTGGAACCATTGATAACTCAGTTCCCCAACATAGATGATGTTGTCTCCTGGGGGCAGCTGCATCTCTAT
GAAGCCACCTCCATTGTTTTCAATTGTTGGGTTTGGCGTTATCAGCATGGCCACGTTCACATCTGGAGATCCAT
GTGCCACTGCCCTGACTGGGATCCTACAGGGCTTTGTTCCAGAGAATGTGACTTCCATGACCACTGTATCATGC
CCACTGTCTGTTGGAGCTCTCTTCCATGTGAACTTTGTTTTGTCACACATTGTGTACGTAAGACCTTTCATCTTCA
GTTTTTCCAGTCCCACTTCGCAGGTCACATGGCCACTCTTCAGGTGGTACTTGGTTCCCTCAATGTGTGCCACAG
GAACCCCAGCGAGAGCCTTCAGTAACACTCCAGTCTGGTCTCCGAGTGCGTACACATCCATCTTGACAGCATGA
GGAGCCCCAAATTCAACCAATCTTTCTGCGTTATTCCAGTTTCGCGCTCCCTCATGTTTCCATGGCAGAGCCAGA
TCATTAAACCAGTCCCTGTGGACCTGCCAAGCCGTTGGAAGGTGTTCCACTGTCTTGTCAAGCTCAAGGATGAC
GGTCTGGGCCAAGTCAACGCCACTAGCGACCCTACACAACAGAGACACATCTCCATACTCACCCATAGTCAGA
ATGGTTTTCTCTGAAGAAACTGTGAAGGATGCCGTCTTCCTCCCACTATGTGTCTCGTTTGCGGCAACATAGTCT
CCCGTGGGG

>1A-prM_2C-primer_EF70119101

GACGTGAGGAAGAAGGGACGGCTCACTGTGATCAGAGCTGAAGGAAAGGACGCAGCAACTCAGGTGCGTGT
GGAGAATGGCACCTGTGTGATCCTGGCTACTGACATGGGGTCATGGTGTGATGATTCACTGTCCTATGAGTGT
GTGACCATAGATCAAGGAGAAGAGCCTGTTGACGTGGATTGTTTTTGCCGGAATGTTGATGGAGTCTATCTGG
AGTATGGACGCTGTGGGGGTCAGCGTGGCTCACGGACAAGGCGCTCAGTGCTGATCCCATCCCATGCCCAGG
GAGAGCTGACGGGGAGGGGACACAAATGGCTAGAAGGAGACTCGCTGCGAACACATCTCACCAGAGTTGAG
GGATGGGTTTGGAAGAACAGGCTACTTGCCCTGGCGATGGTCACCGTTGTGTGGTTGACCCTGGAGAGTGTG
GTGACCAGGGTCGCCGTTCTGGTTGTGCTCCTGTGTTTGGCGCCGGTCTACGCTTCGCGTTGCACACATTTGGA
AAACAGGGACTTTGTGACTGGTACTCAGGGGACTACGAGGGTCACCTTGGTGCTGGAACTGGGTGGATGTGT
TACCATAACAGCTGAGGGGAAGCCTCAG

>1A-prM_2D-primer_EF70119102

TGGTACCATCCACCCAGTTCCAGCACCAAGGTGACCCTCGTAGTCCCCTGAGTACCAGTCACAAAGTCCCTGTT
TTCCAAATGTGTGCAACGCGAAGCGTAGACCGGCGCCAAACACAGGAGCACAACCAGAACGGCGACCCTGGT
CACCACACTCTCCAGGGTCAACCACACAACGGTGACCATCGCCAGGGCAAGTAGCCTGTTCTTCCAAACCCATC
CCTCAACTCTGGTGAGATGTGTTCGCAGCGAGTCTCCTTCTAGCCATTTGTGTCCCCTCCCCGTCAGCTCTCCCT
GGGCATGGGATGGGATCAGCACTGAGCGCCTTGTCCGTGAGCCACGCTGACCCCCACAGCGTCCATACTCCAG
ATAGACTCCATCAACATTCCGGCAAAAACAATCCACGTCAACAGGCTCTTCTCCTTGATCTATGGTCACACACTC
ATAGGACAGTGAATCATCACACCATGACCCCATGTCAGTAGCCAGGATCACACAGGTGCCATTCTCCACACGC
ACCTGAGTTGCTGCGTCCTTTCCTTCAGCTCTGATCACAGTTGAGCCGTCCCTTTCTTTCCTCACGGTTGCAGCA
ATCGTCATCCCCAACAAGAT

Supplementary Table 2 | Details of the Molecular Dynamics simulations. The table reports from left to right: the name of the system, the number of lipids per each species, the number of potassium and

chloride ions in solution, the number of water molecules, the total number of atoms, and the production simulation time.

System	# Lipids					# K/Cl ions	# waters	Total # atoms	Production time [µs]
	POPC	POPE	POPS	POPI	CHOL				
$(prM\text{-}E)_2^{WT}$	444	185	37	37	37	258/184	67294	317505	1.0
$(prM\text{-}E)_2^{MUT}$	444	185	37	37	37	257/185	67846	319141	1.0
$(sE)_2^{WT}$	-	-	-	-	-	85/81	25308	88114	1.0
$(sE)_2^{MUT}$	-	-	-	-	-	84/80	24793	86559	1.0
