# Supplementary material for: Irreversible furin cleavage site exposure renders immature tick-borne flaviviruses fully infectious
Source: Nat Commun. 2025 Aug 12;16:7491. doi: 10.1038/s41467-025-62750-6 (PMC12343913; doi:10.1038/s41467-025-62750-6)
Supplement: Supplementary file 2 — Reporting summary [file 41467_2025_62750_MOESM2_ESM.pdf]

nature portfolio

Corresponding author(s): Daniel Ruzek, Félix A. Rey

Last updated by author(s): Jul 17, 2025

# Reporting Summary

Nature Portfolio wishes to improve the reproducibility of the work that we publish. This form provides structure for consistency and transparency in reporting. For further information on Nature Portfolio policies, see our Editorial Policies and the Editorial Policy Checklist.

## Statistics

For all statistical analyses, confirm that the following items are present in the figure legend, table legend, main text, or Methods section.

| n/a | Confirmed | |
|---|---|---|
| ☐ | ☒ | The exact sample size (*n*) for each experimental group/condition, given as a discrete number and unit of measurement |
| ☐ | ☒ | A statement on whether measurements were taken from distinct samples or whether the same sample was measured repeatedly |
| ☐ | ☒ | The statistical test(s) used AND whether they are one- or two-sided<br>*Only common tests should be described solely by name; describe more complex techniques in the Methods section.* |
| ☒ | ☐ | A description of all covariates tested |
| ☒ | ☐ | A description of any assumptions or corrections, such as tests of normality and adjustment for multiple comparisons |
| ☐ | ☒ | A full description of the statistical parameters including central tendency (e.g. means) or other basic estimates (e.g. regression coefficient) AND variation (e.g. standard deviation) or associated estimates of uncertainty (e.g. confidence intervals) |
| ☐ | ☒ | For null hypothesis testing, the test statistic (e.g. *F*, *t*, *r*) with confidence intervals, effect sizes, degrees of freedom and *P* value noted<br>*Give P values as exact values whenever suitable.* |
| ☒ | ☐ | For Bayesian analysis, information on the choice of priors and Markov chain Monte Carlo settings |
| ☒ | ☐ | For hierarchical and complex designs, identification of the appropriate level for tests and full reporting of outcomes |
| ☒ | ☐ | Estimates of effect sizes (e.g. Cohen's *d*, Pearson's *r*), indicating how they were calculated |

*Our web collection on statistics for biologists contains articles on many of the points above.*

## Software and code

Policy information about availability of computer code

| | |
|---|---|
| Data collection | LightCycler 480 Software, Version 1.5 (Roche)<br>Olympus Xcellence software for aquisition of microscopy images.<br>ImageXpress software for aquisition and analysis of microscopy images.<br>Raw images from microscope were processed with ImageJ/Fiji software 1.53h.<br>Enzymatic analysis were collected with Tecan Magellan software.<br>Amersham™ Imager 680 (GE Healthcare). |
| Data analysis | Data were plotted into graphs and statistically analysed using GraphPad Prism software version 8.<br>AlphaFold2 was used for structure predictions<br>Structural analysis of models was performed in PyMOL 2.5.4.<br>Clustal Omega and MAFFT were used for multiple sequence alignments<br>ESPript 3.0 was used for MSA editing<br>MODELLER v. 10.1<br>PPM 3.0 webserver<br>CHARMM-GUI server<br>GROMACS v. 2022.5<br>MDAnalysis v.2.3.0 |

For manuscripts utilizing custom algorithms or software that are central to the research but not yet described in published literature, software must be made available to editors and reviewers. We strongly encourage code deposition in a community repository (e.g. GitHub). See the Nature Portfolio guidelines for submitting code & software for further information.

# Data

Policy information about availability of data

All manuscripts must include a data availability statement. This statement should provide the following information, where applicable:

- Accession codes, unique identifiers, or web links for publicly available datasets
- A description of any restrictions on data availability
- For clinical datasets or third party data, please ensure that the statement adheres to our policy

The authors declare that data supporting the findings of this study are available within the paper and its supplementary informations file + Source Data File. All other data and explanations are available from the corresponding author upon reasonable request. Published data were taken from GenBank (www.ncbi.nlm.nih.gov/genbank), UniProt (www.uniprot.org) and Protein Data Bank, PDB (www.rcsb.org).

# Research involving human participants, their data, or biological material

Policy information about studies with human participants or human data. See also policy information about sex, gender (identity/presentation), and sexual orientation and race, ethnicity and racism.

| | |
|---|---|
| Reporting on sex and gender | Our research does not involve human paticipants. |
| Reporting on race, ethnicity, or other socially relevant groupings | Our research does not involve human paticipants. |
| Population characteristics | Our research does not involve human paticipants. |
| Recruitment | Our research does not involve human paticipants. |
| Ethics oversight | Our research does not involve human paticipants. |

Note that full information on the approval of the study protocol must also be provided in the manuscript.

# Field-specific reporting

Please select the one below that is the best fit for your research. If you are not sure, read the appropriate sections before making your selection.

☒ Life sciences ☐ Behavioural & social sciences ☐ Ecological, evolutionary & environmental sciences

For a reference copy of the document with all sections, see nature.com/documents/nr-reporting-summary-flat.pdf

# Life sciences study design

All studies must disclose on these points even when the disclosure is negative.

| | |
|---|---|
| Sample size | No sample-size calculation was performed. The sample sizes were chosen based on experience and minimal number of replicates needed for the statistical evaluation. |
| Data exclusions | No data were excluded. |
| Replication | Experiments successfully repeated at least twice. |
| Randomization | he mice were randomly assigned to cages and the cages were then randomized into groups. |
| Blinding | Blinding was not relevant to this study. The readouts of all experiments could be assessed objectively. |

# Reporting for specific materials, systems and methods

We require information from authors about some types of materials, experimental systems and methods used in many studies. Here, indicate whether each material, system or method listed is relevant to your study. If you are not sure if a list item applies to your research, read the appropriate section before selecting a response.

## Materials & experimental systems

| n/a | Involved in the study |
|---|---|
| ☐ | ☒ Antibodies |
| ☐ | ☒ Eukaryotic cell lines |
| ☒ | ☐ Palaeontology and archaeology |
| ☐ | ☒ Animals and other organisms |
| ☒ | ☐ Clinical data |
| ☒ | ☐ Dual use research of concern |
| ☒ | ☐ Plants |

## Methods

| n/a | Involved in the study |
|---|---|
| ☒ | ☐ ChIP-seq |
| ☒ | ☐ Flow cytometry |
| ☒ | ☐ MRI-based neuroimaging |

# Antibodies

Antibodies used

TBEV E protein (mouse monoclonal antibody 1493,diluted 1:2000; PMID:7817895)
TBEV M protein (in-house rabbit polyclonal serum, diluted 1:500)
Anti-flavivirus E protein 4G2 antibody (Merck, MAB10216-I-100UG; diluted 1:250)
anti-mouse IgG-HRP (Invitrogen AB_228307)
anti-rabbit IgG-HRP (Invitrogen AB_228341)

Validation

Anti-TBEV antibodies were previously reported and validated (PMID:7817895 and 35458522).
No validation statements for the other antibodies that are commercially available.

# Eukaryotic cell lines

Policy information about cell lines and Sex and Gender in Research

Cell line source(s)

BHK-21 (ATTC CCL-10)
Vero cells (ATCC CCL-81)
LoVo cells (ATCC CCL-229)
Porcine kidney stable cells - PS (provided by the National Reference Laboratory for Cell Cultures, National Institute of Public Health, Prague)
Tick cell line IRE/CTVM19 (derived from Ixodes ricinus, provided by the Tick Cell Biobank, University of Liverpool, UK)

Authentication

Not authenticated after purchase.

Mycoplasma contamination

In situ analysis never detected Mycoplasma infection.

Commonly misidentified lines
(See ICLAC register)

No commonly misidentified cell lines have been used in this study.

# Animals and other research organisms

Policy information about studies involving animals; ARRIVE guidelines recommended for reporting animal research, and Sex and Gender in Research

Laboratory animals

6-week-old BALB/c female mice (ENVIGO RMS) were housed in individually ventilated cages in the BSL-3 animal facility with controlled environment with a 12/12 hour light-dark cycle. Animals were fed the standard chow diet ad libitum. Water bottles with fresh drinking water were changed twice a week.

Wild animals

The study did not involve wild animals.

Reporting on sex

Only females were used in this study.

Field-collected samples

The study did not involve field-collected samples.

Ethics oversight

The animal experiments complied with all relevant European Union guidelines for work with animals and were carried out in accordance with Czech national law guidelines on the use of experimental animals and protection of animals against cruelty (Animal Welfare Act No. 246/1992 Coll.). The protocol was approved by the Committee on the Ethics of Animal Experimentation of the Institute of Parasitology and the Departmental Expert Committee for the Approval of Projects of Experiments on Animals of the Czech Academy of Sciences (permit no. 111/2020).

Note that full information on the approval of the study protocol must also be provided in the manuscript.

## Plants

| | |
|---|---|
| Seed stocks | Our study does not involve plants or seeds. |
| Novel plant genotypes | Our study does not involve plants or seeds. |
| Authentication | Our study does not involve plants or seeds. |
