# Supplementary figures and images for: Irreversible furin cleavage site exposure renders immature tick-borne flaviviruses fully infectious
Source: Nat Commun. 2025 Aug 12;16:7491. doi: 10.1038/s41467-025-62750-6 (PMC12343913; doi:10.1038/s41467-025-62750-6)

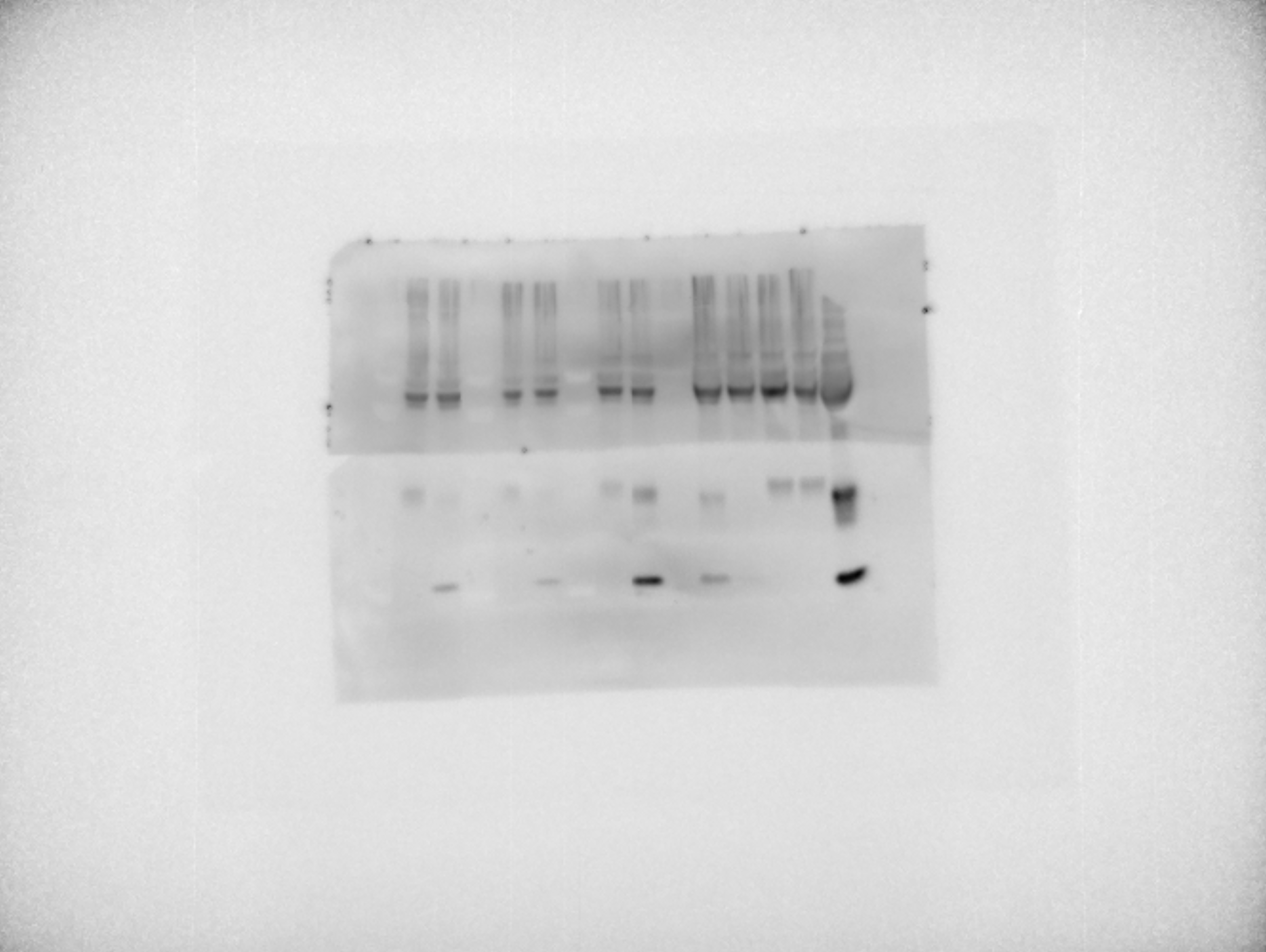

Supplement: Supplementary file 4 — Source Data [file 41467_2025_62750_MOESM4_ESM.zip › SourceDataFiles/images/2024-0425__immatuire_ver003_fig1_FromLeft_lines_M-1-2_.tif]

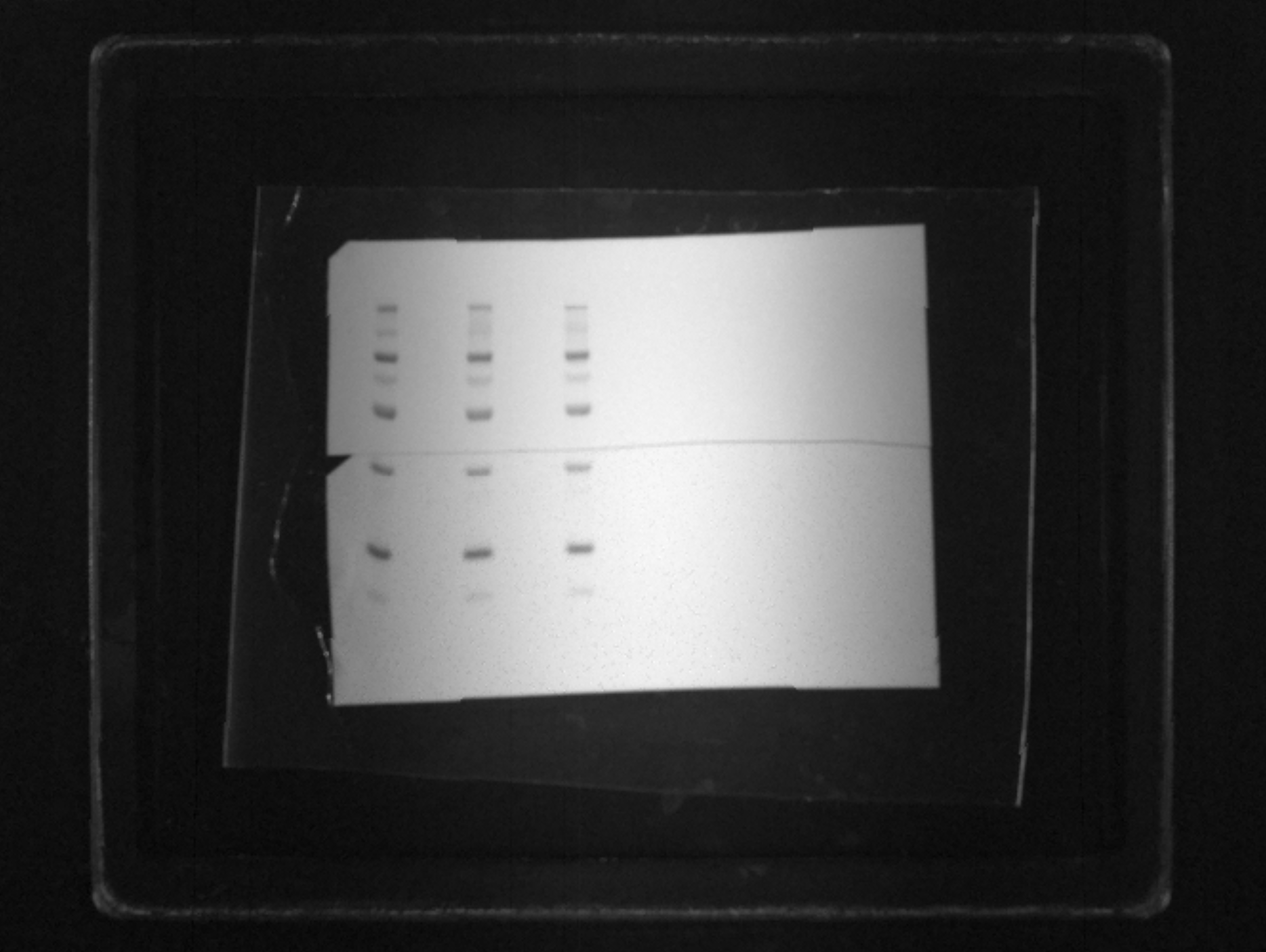

Supplement: Supplementary file 4 — Source Data [file 41467_2025_62750_MOESM4_ESM.zip › SourceDataFiles/images/2024-0425__immatuire_ver003_fig1_FromLeft_lines_M-1-2_MarkerOnly.tif]

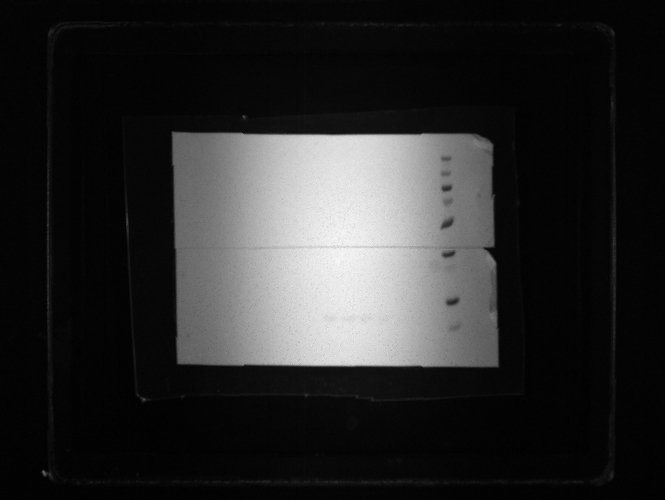

Supplement: Supplementary file 4 — Source Data [file 41467_2025_62750_MOESM4_ESM.zip › SourceDataFiles/images/2024-0531-142337_SupplementFig2_.jpg]

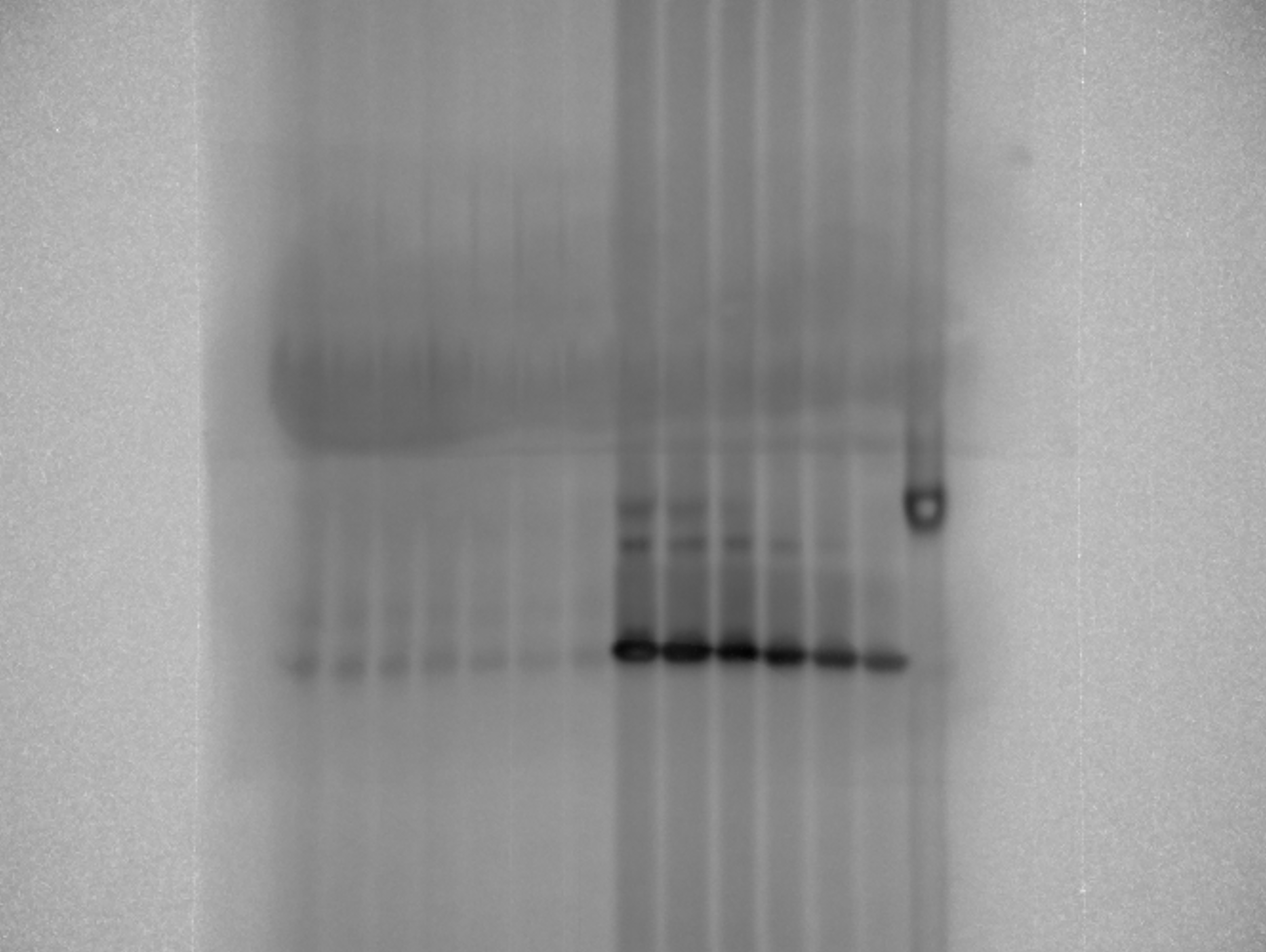

Supplement: Supplementary file 4 — Source Data [file 41467_2025_62750_MOESM4_ESM.zip › SourceDataFiles/images/2024-0531-142337_SupplementFig2_MarkerOnly.tif]

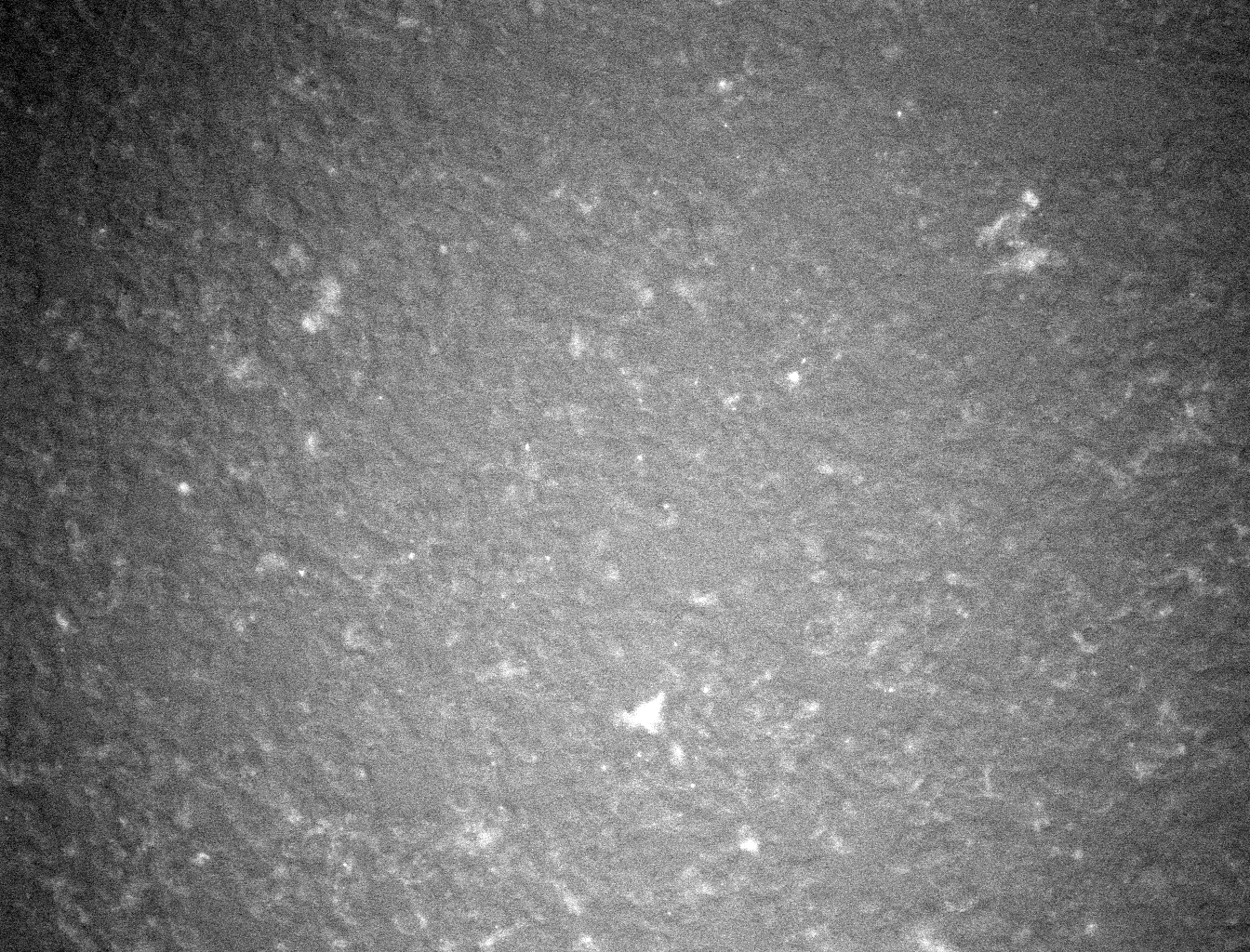

Supplement: Supplementary file 4 — Source Data [file 41467_2025_62750_MOESM4_ESM.zip › SourceDataFiles/images/Fig1f_images/prM_TBEV_ LOVO_cells.tif]

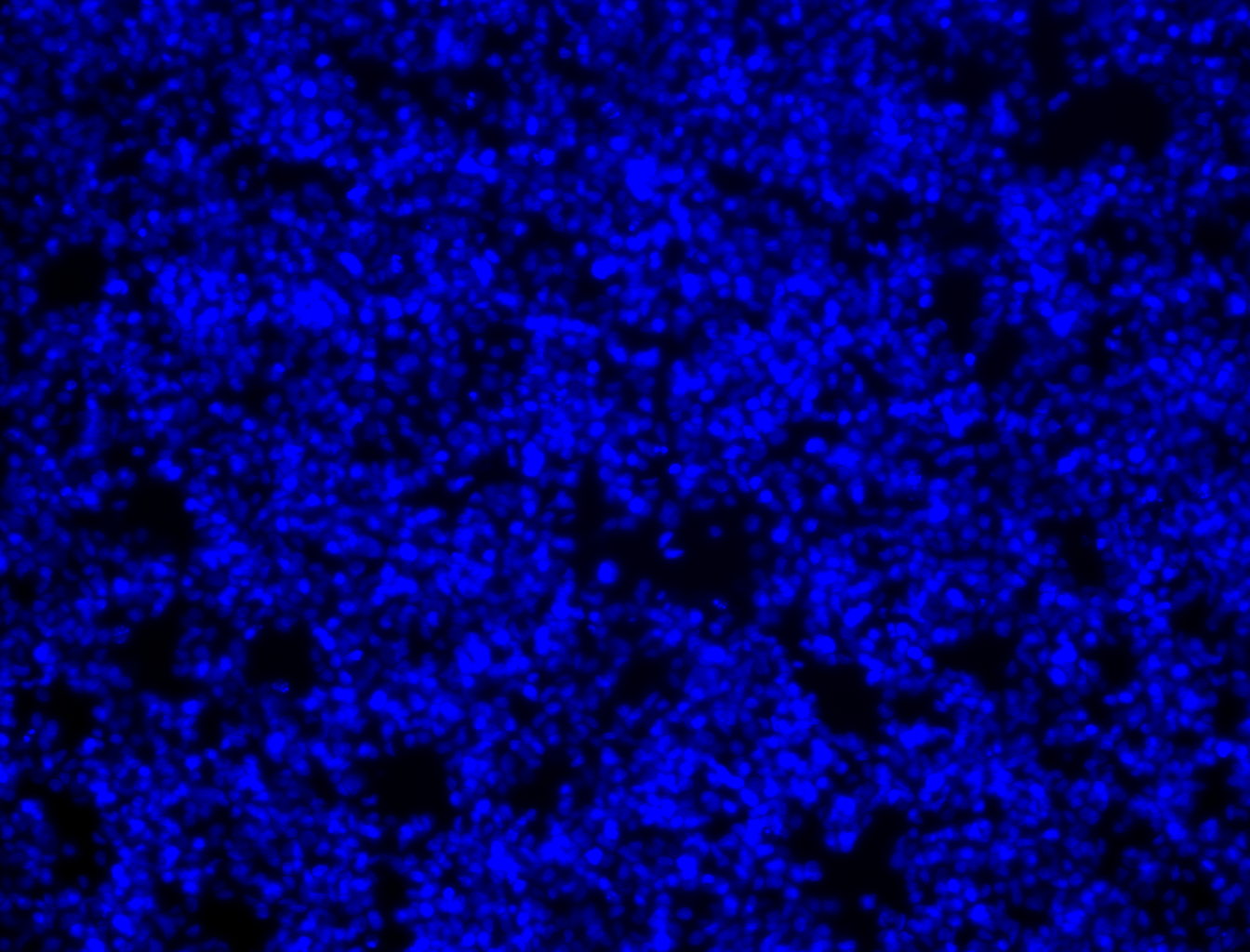

Supplement: Supplementary file 4 — Source Data [file 41467_2025_62750_MOESM4_ESM.zip › SourceDataFiles/images/Fig1f_images/prM_TBEV_moi=1_72hpi_mcherry_on_LoVo_merged.png]

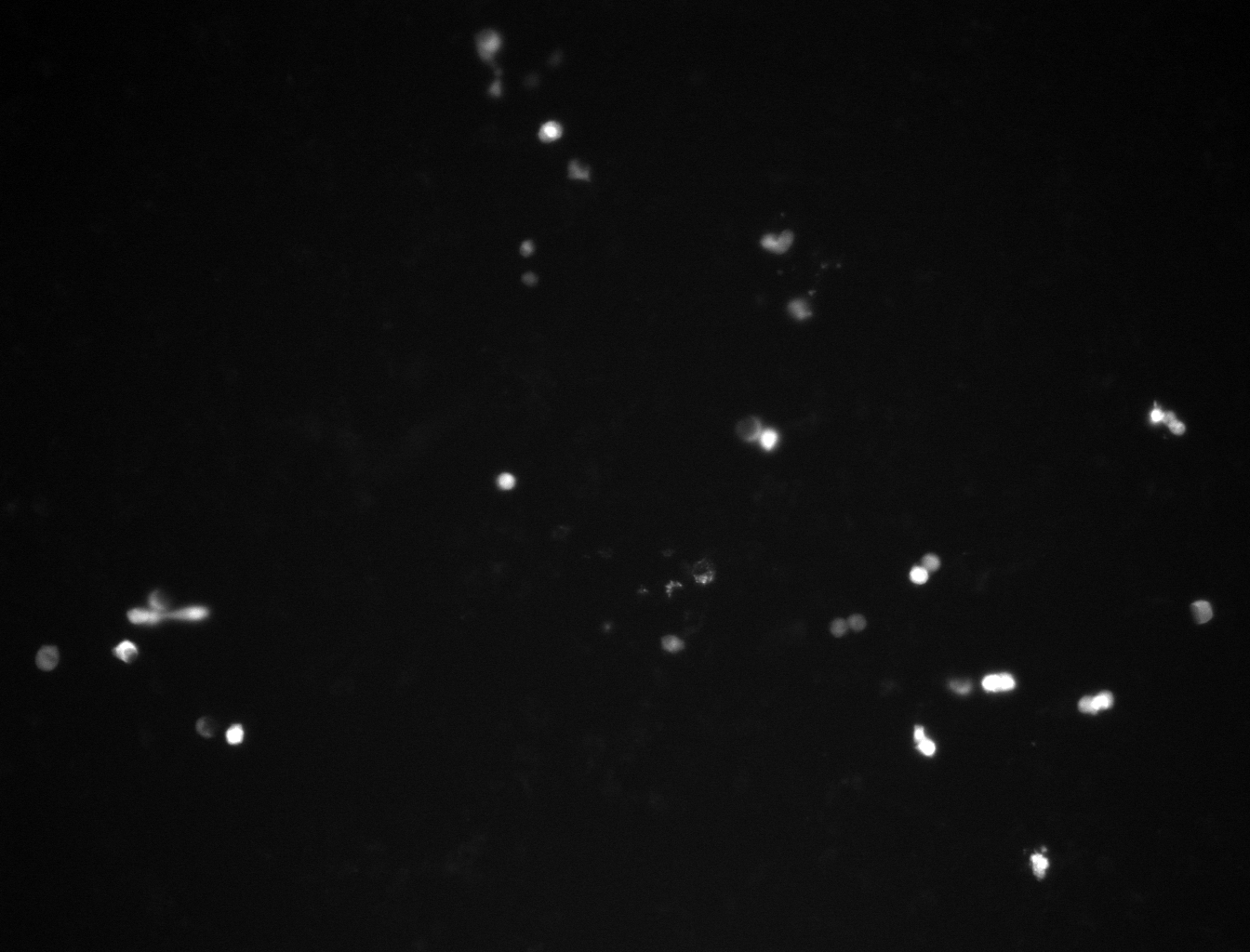

Supplement: Supplementary file 4 — Source Data [file 41467_2025_62750_MOESM4_ESM.zip › SourceDataFiles/images/Fig1f_images/WT_TBEV_LOVO_cells.tif]

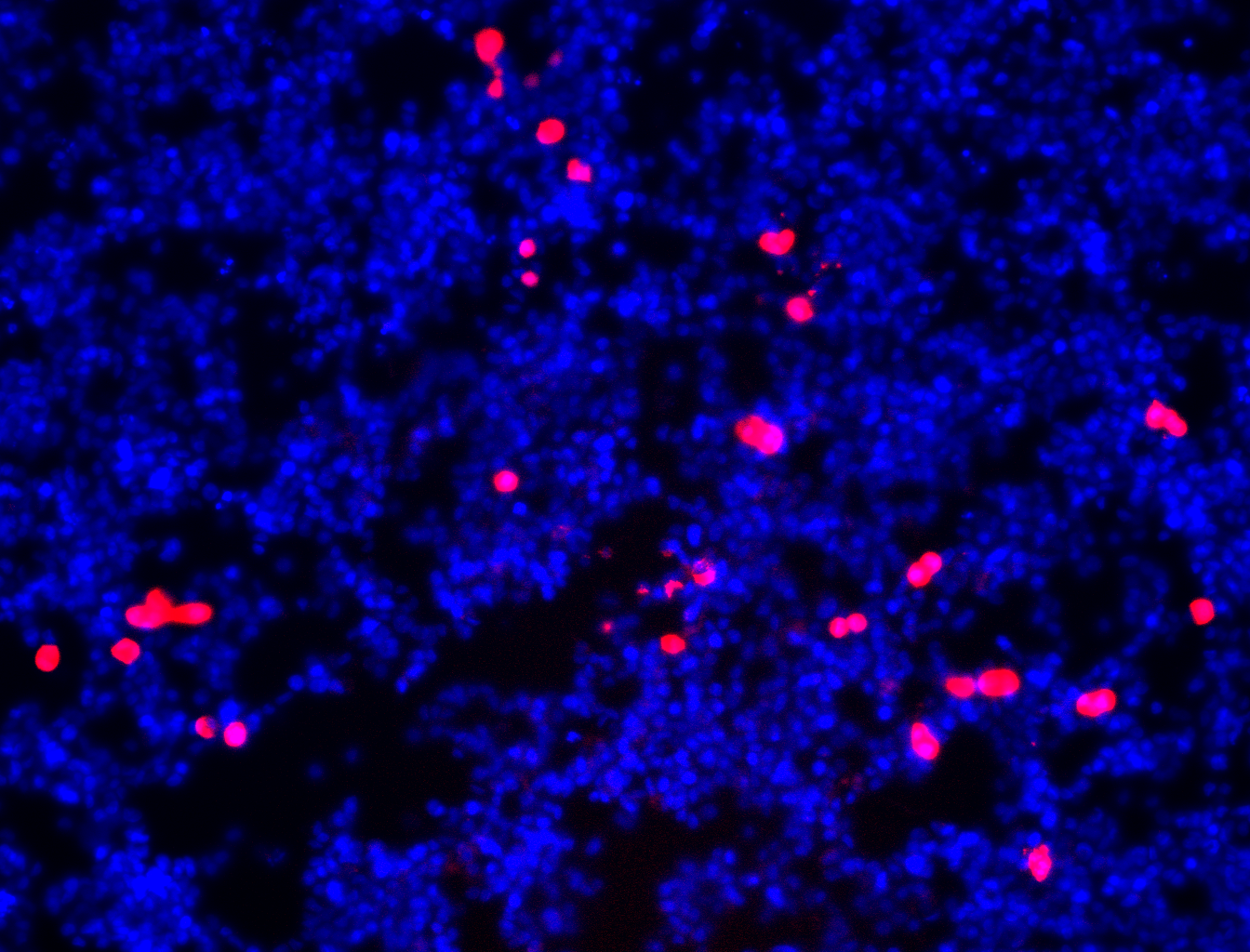

Supplement: Supplementary file 4 — Source Data [file 41467_2025_62750_MOESM4_ESM.zip › SourceDataFiles/images/Fig1f_images/WT_TBEV_moi=1_72hpi_mcherry_on_LoVo_merged.png]

## Slide 1
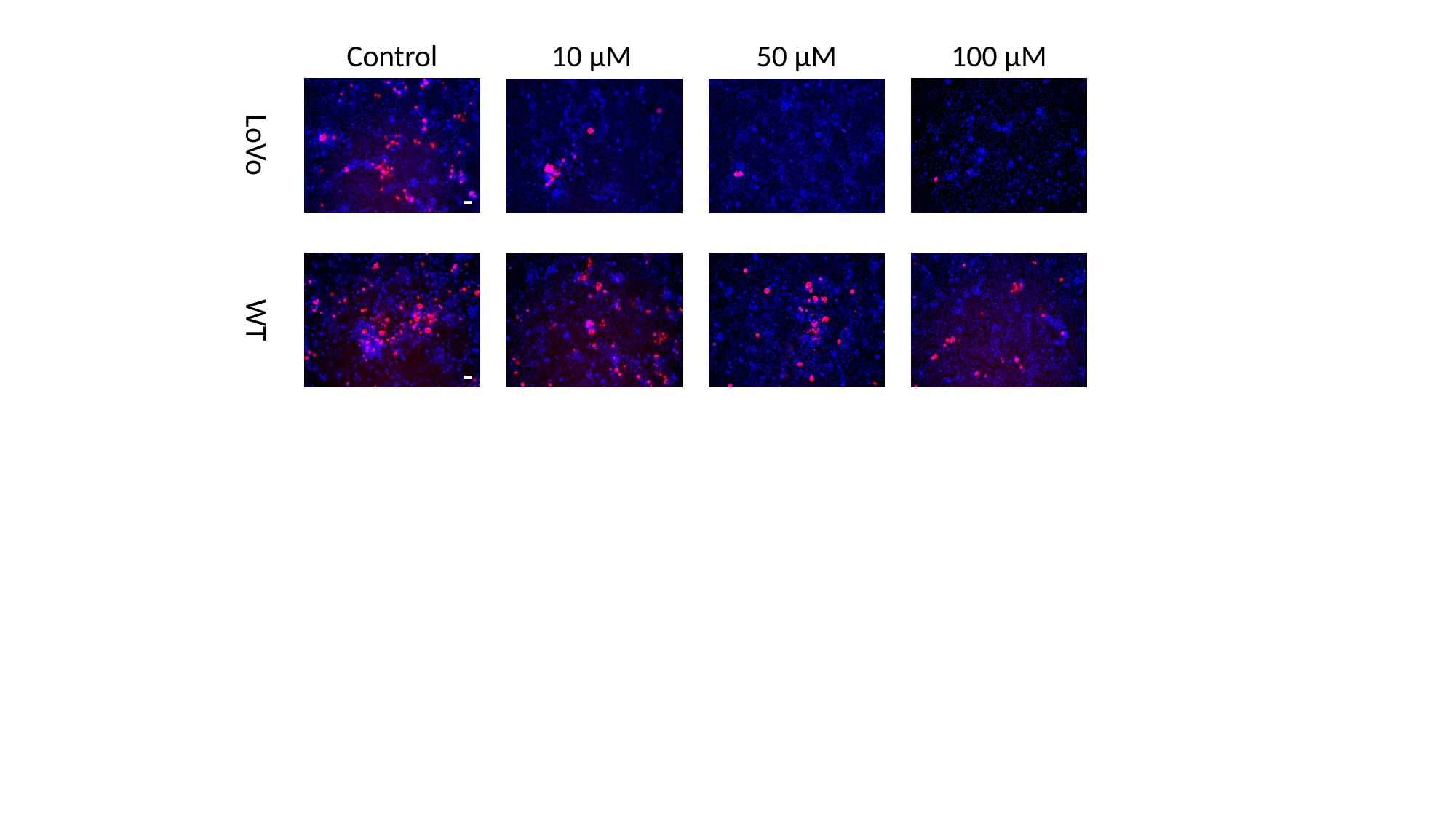

Control
10 µM
50 µM
100 µM
LoVo
WT

Supplement: Supplementary file 4 — Source Data [file 41467_2025_62750_MOESM4_ESM.zip › SourceDataFiles/images/Fig1h_images/Furin_inhibitor_CMK_graph_&_mCherry_Fig-1h.pptx]

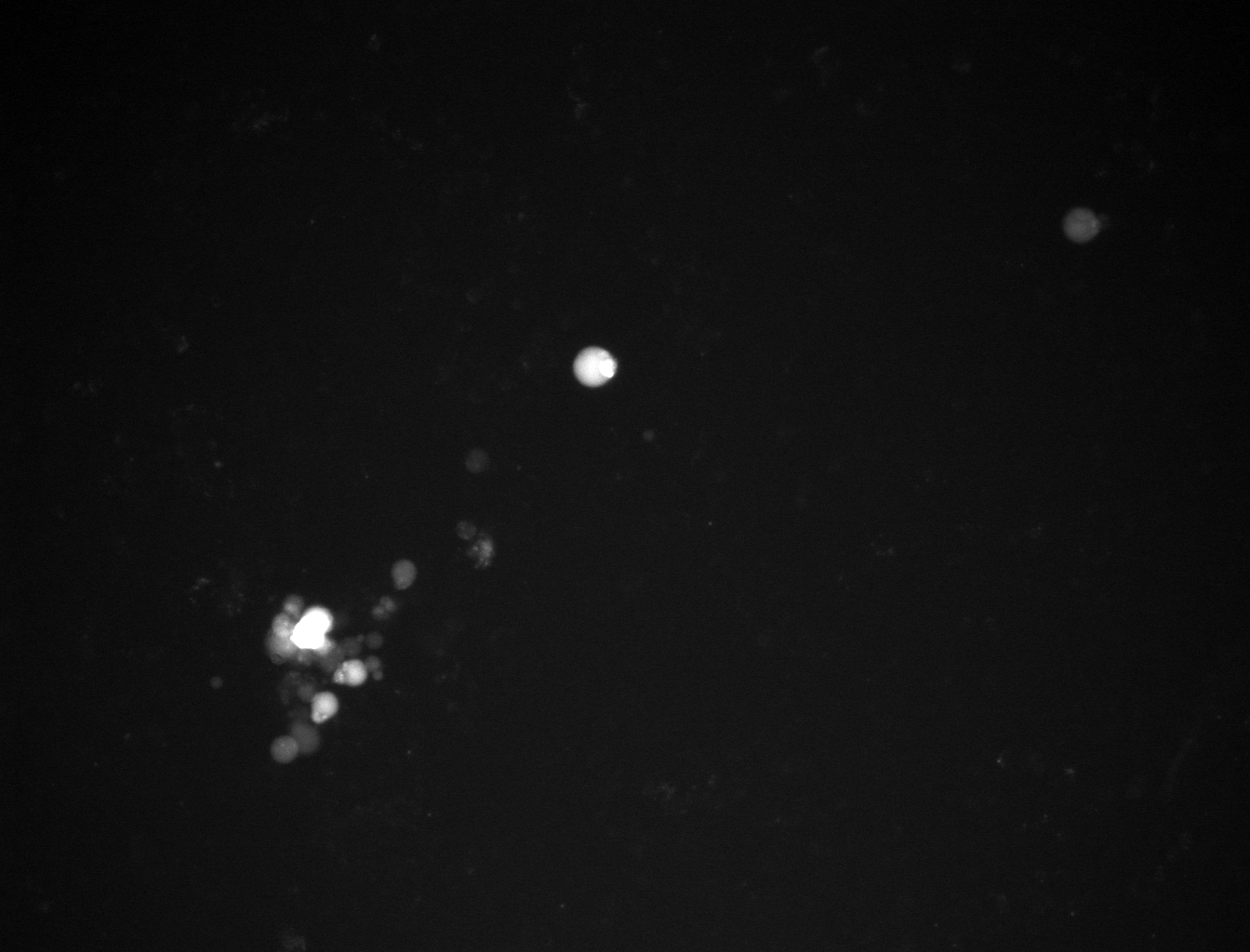

Supplement: Supplementary file 4 — Source Data [file 41467_2025_62750_MOESM4_ESM.zip › SourceDataFiles/images/Fig1h_images/Lovo 10/D85VQ8LC_F00002533.tif]

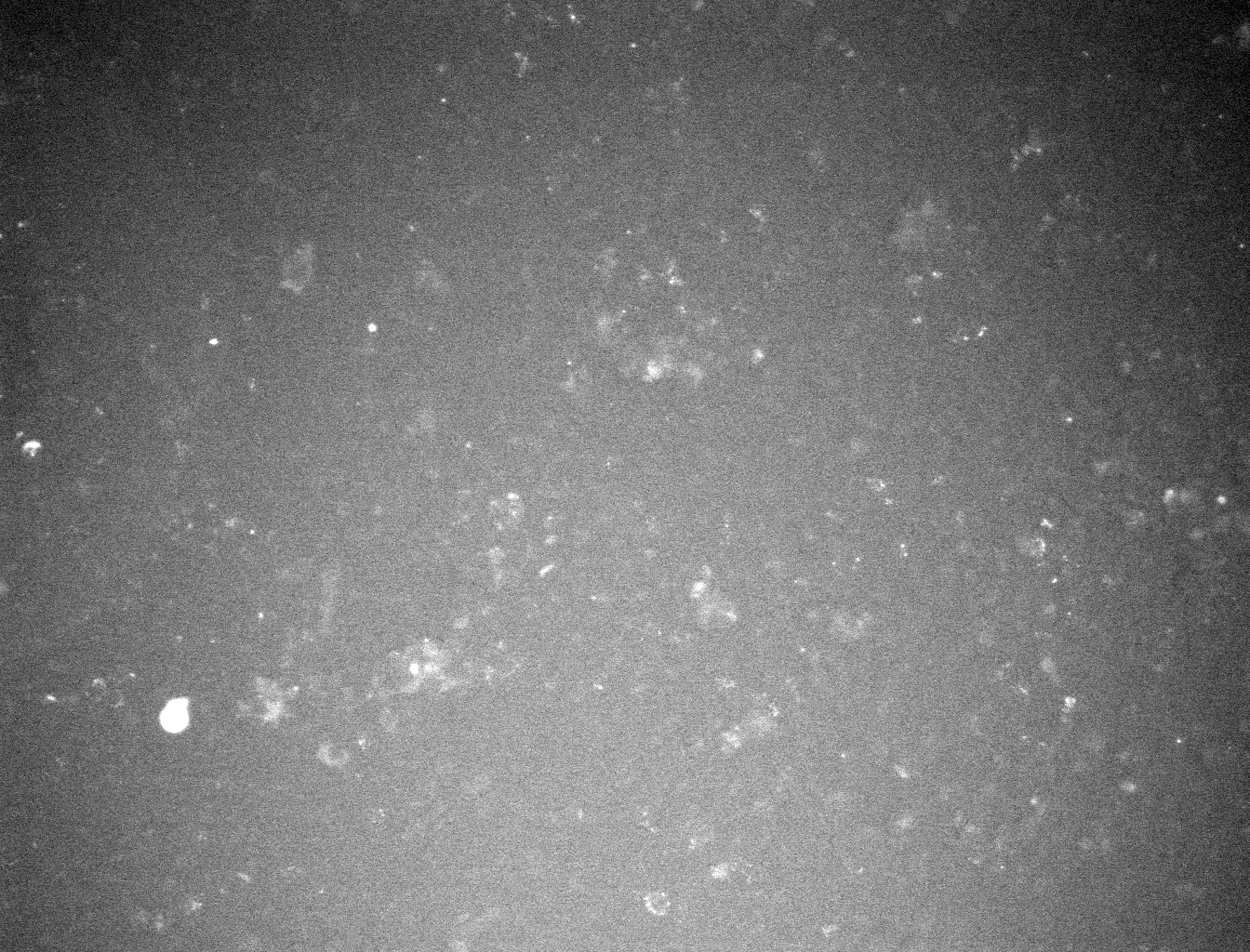

Supplement: Supplementary file 4 — Source Data [file 41467_2025_62750_MOESM4_ESM.zip › SourceDataFiles/images/Fig1h_images/lovo 100/D85VQ8LC_F00002542.tif]

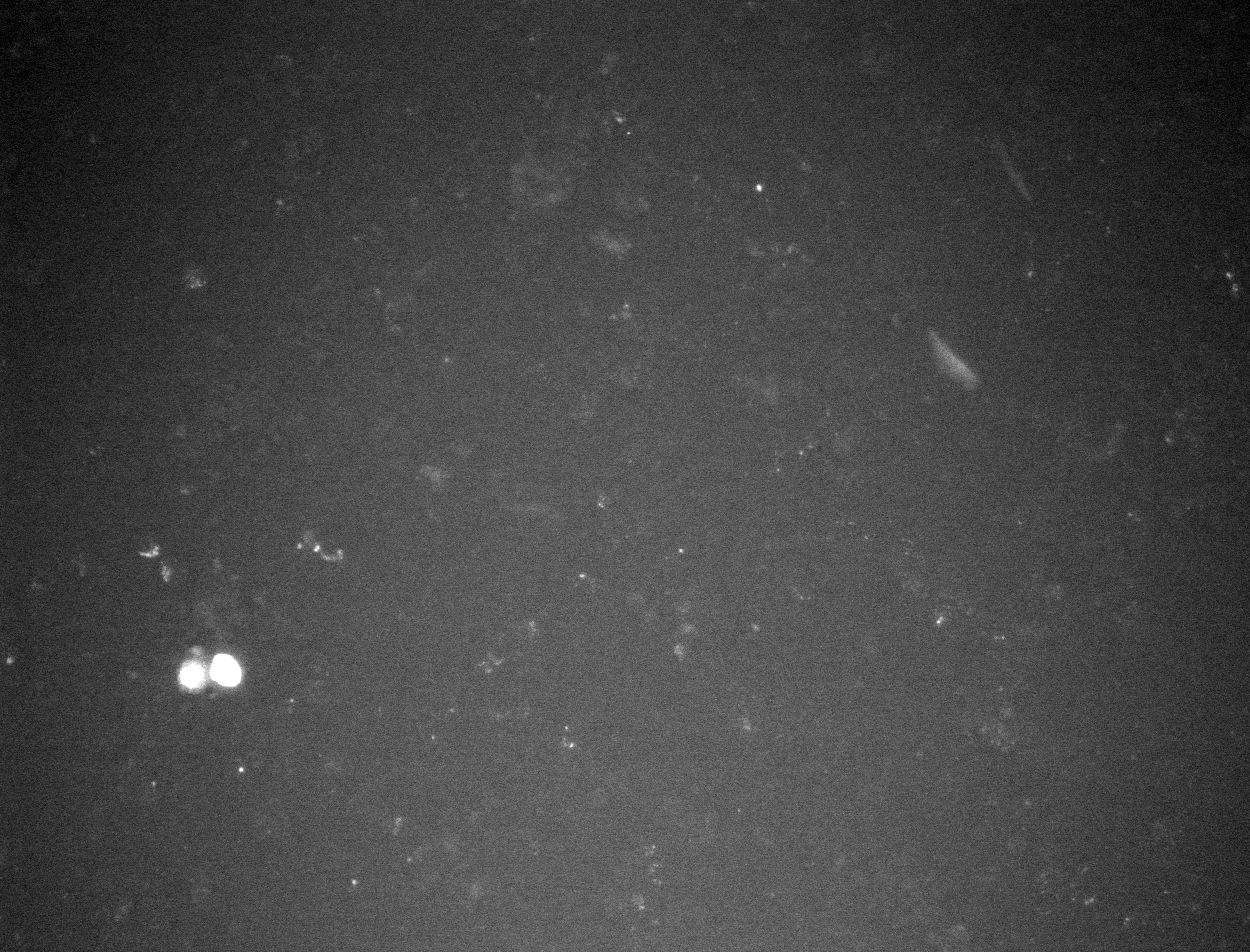

Supplement: Supplementary file 4 — Source Data [file 41467_2025_62750_MOESM4_ESM.zip › SourceDataFiles/images/Fig1h_images/Lovo 50/D85VQ8LC_F00002536.tif]

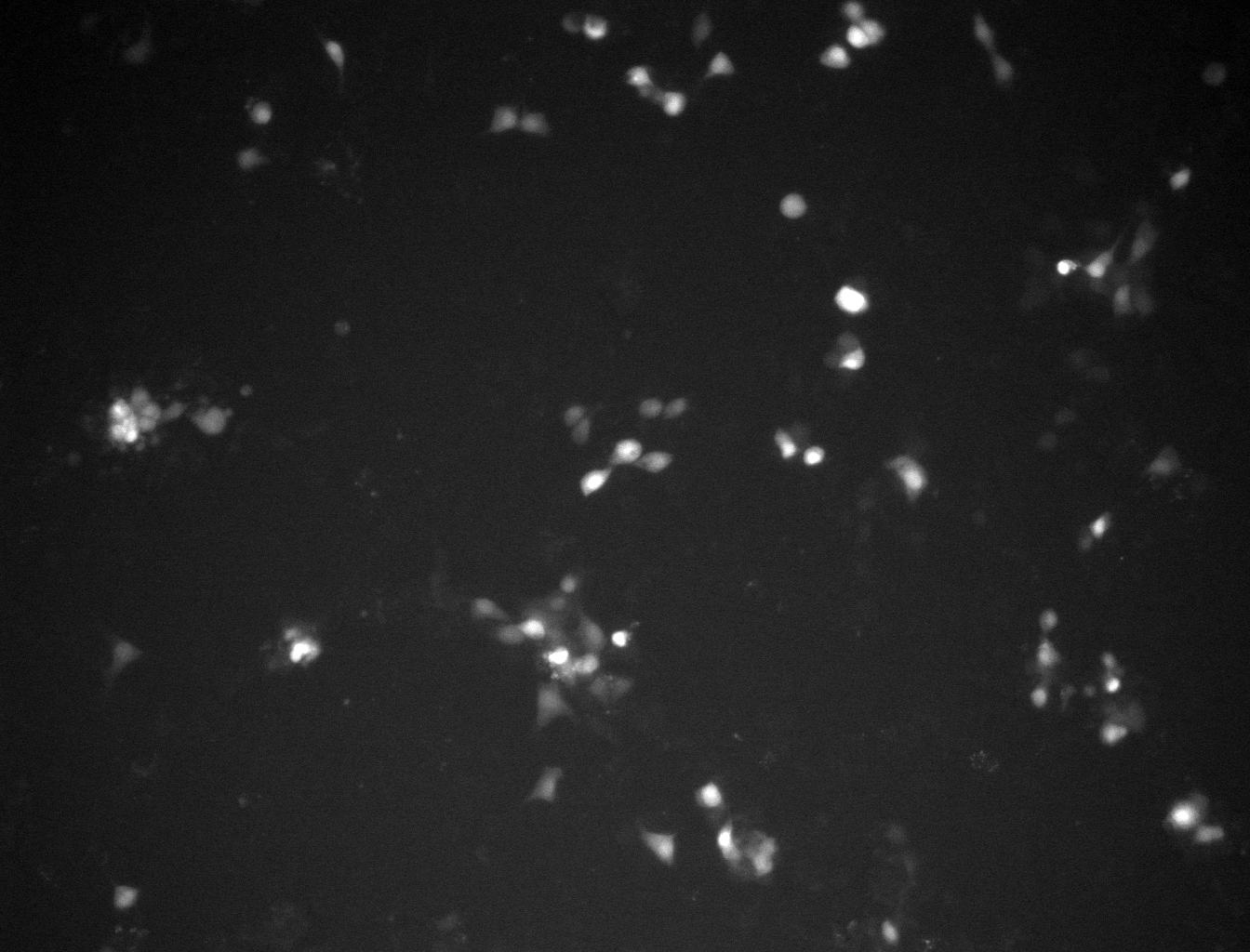

Supplement: Supplementary file 4 — Source Data [file 41467_2025_62750_MOESM4_ESM.zip › SourceDataFiles/images/Fig1h_images/Lovo kontrola/D85VQ8LC_F00002530 lepsie.tif]

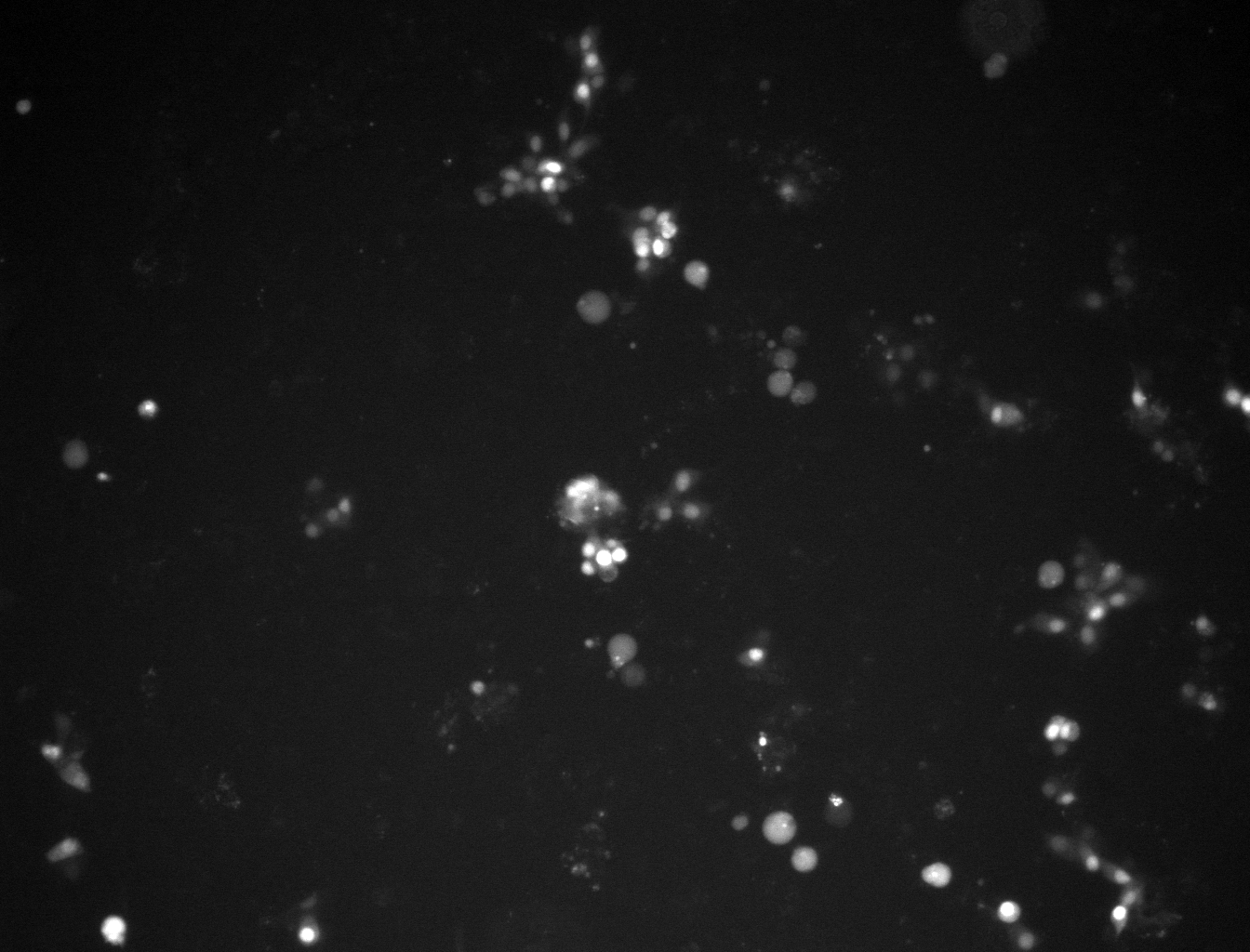

Supplement: Supplementary file 4 — Source Data [file 41467_2025_62750_MOESM4_ESM.zip › SourceDataFiles/images/Fig1h_images/WT 10/D85VQ8LC_F00002548.tif]

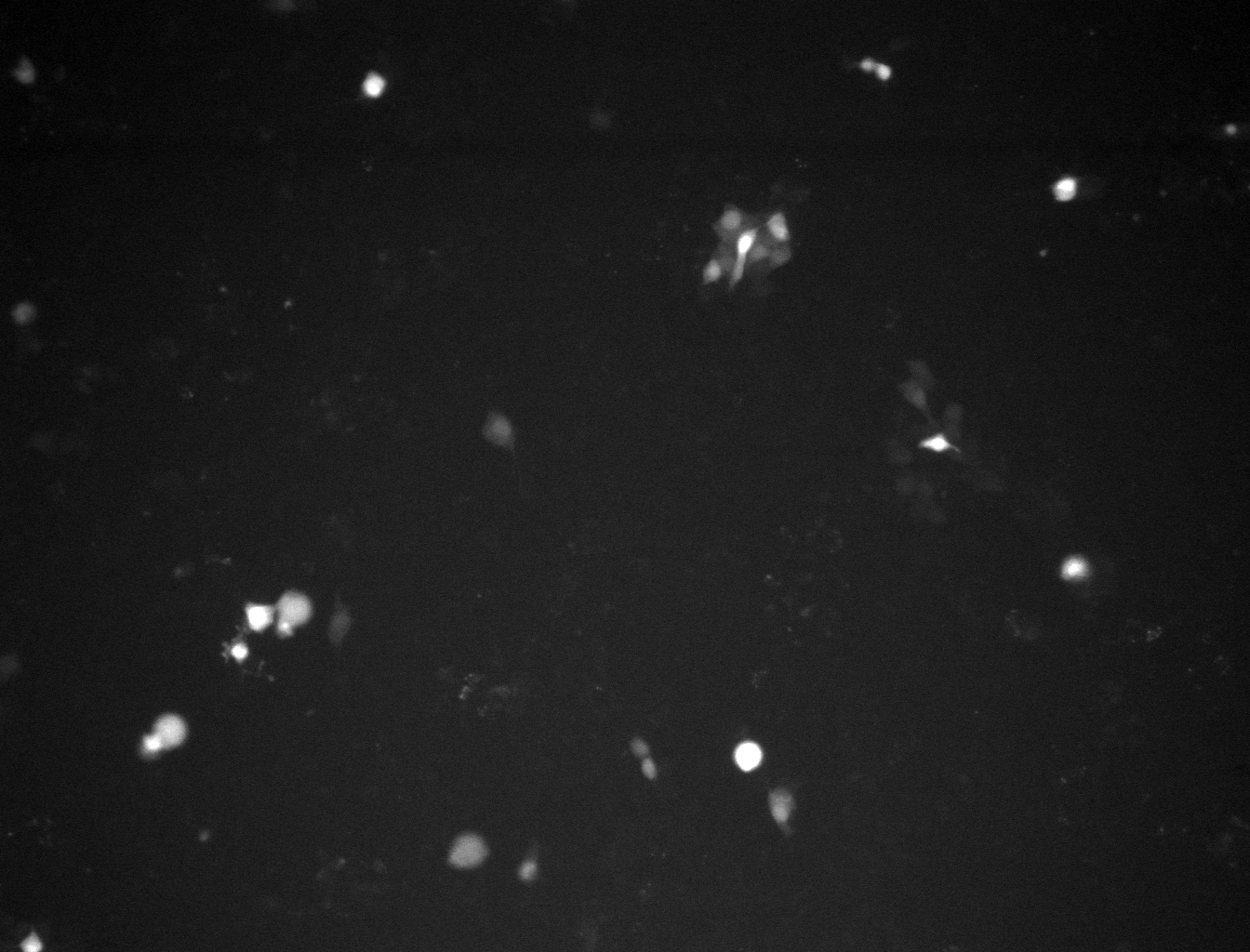

Supplement: Supplementary file 4 — Source Data [file 41467_2025_62750_MOESM4_ESM.zip › SourceDataFiles/images/Fig1h_images/WT 100/D85VQ8LC_F00002554.tif]

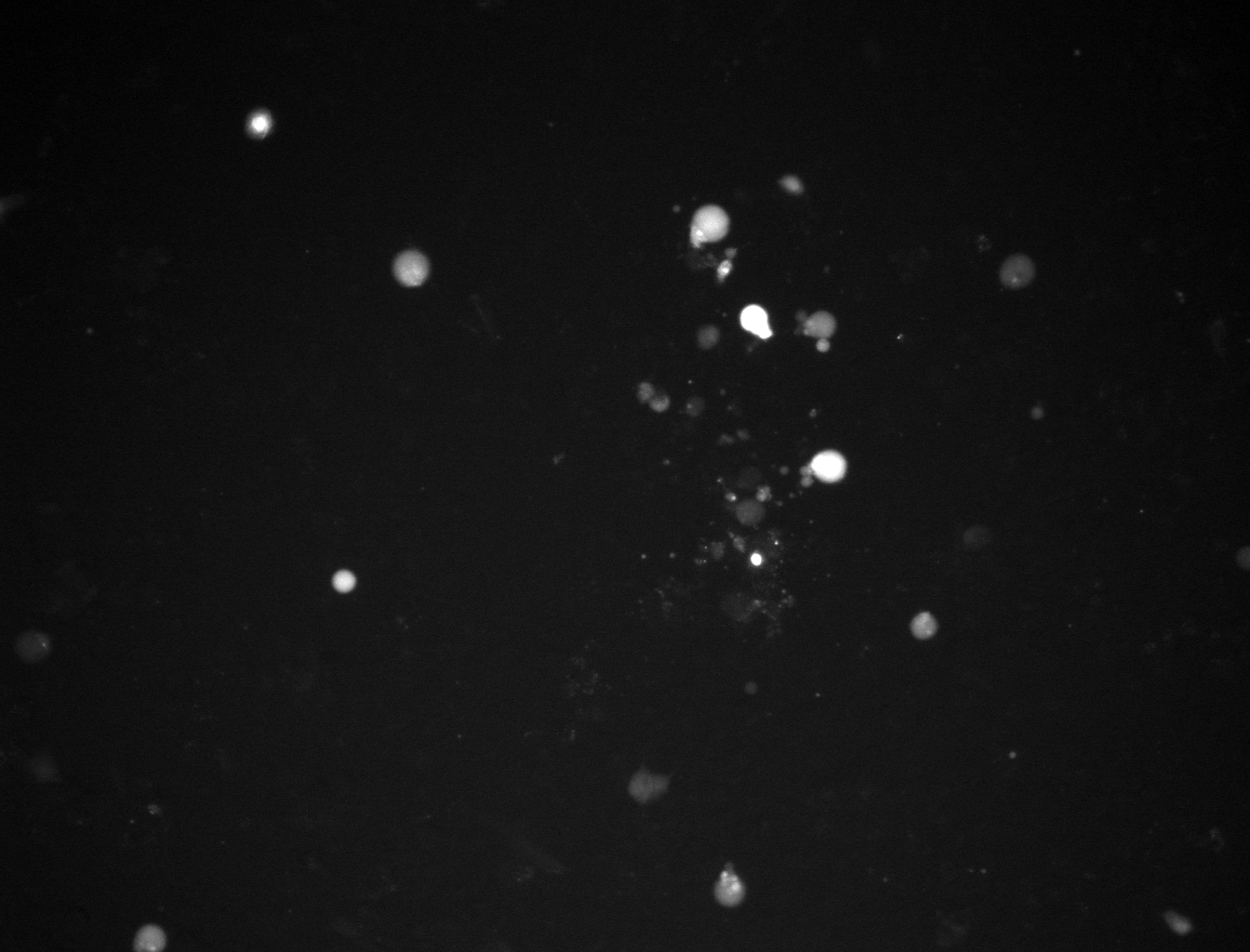

Supplement: Supplementary file 4 — Source Data [file 41467_2025_62750_MOESM4_ESM.zip › SourceDataFiles/images/Fig1h_images/WT 50/D85VQ8LC_F00002551.tif]

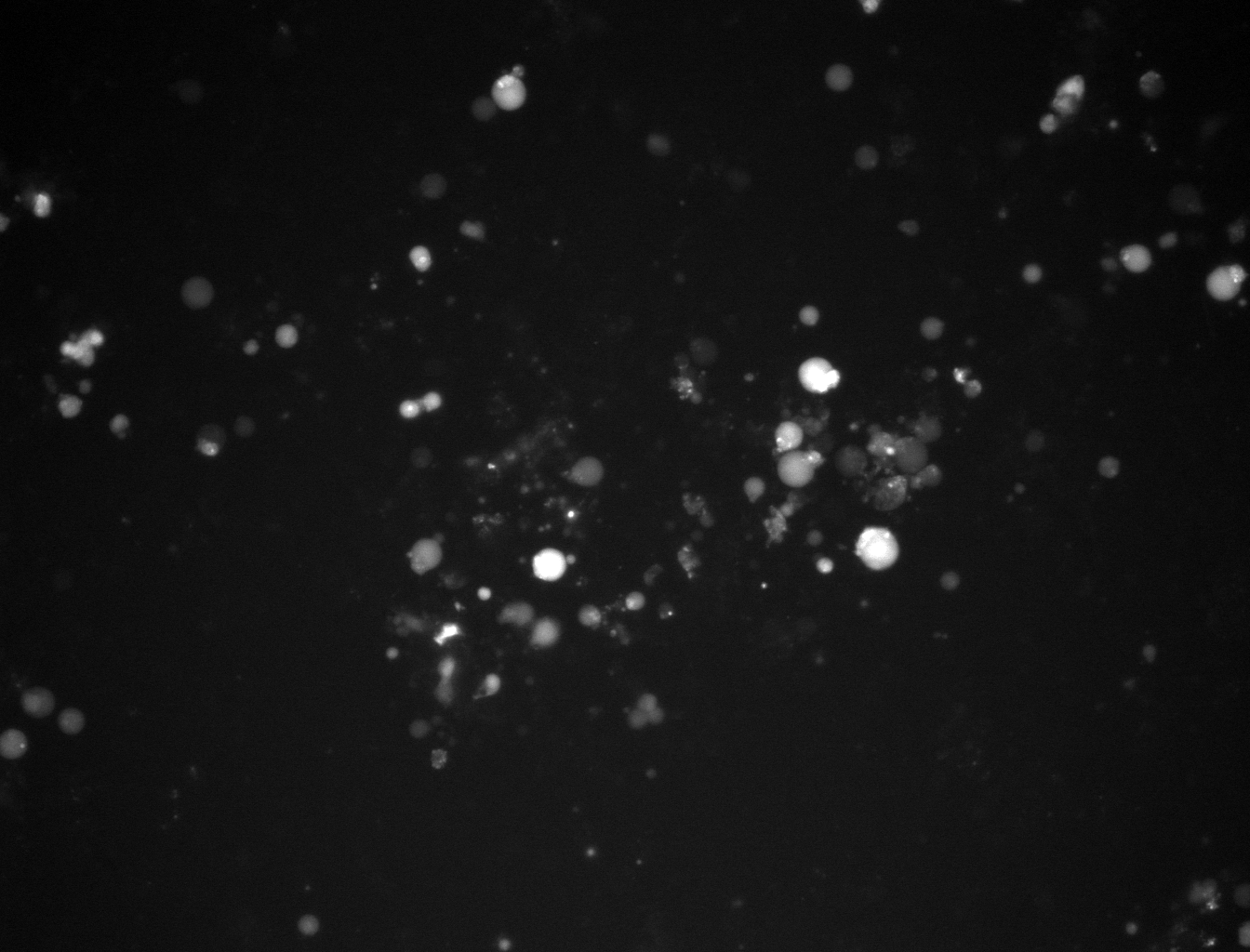

Supplement: Supplementary file 4 — Source Data [file 41467_2025_62750_MOESM4_ESM.zip › SourceDataFiles/images/Fig1h_images/WT kontrola/D85VQ8LC_F00002545.tif]

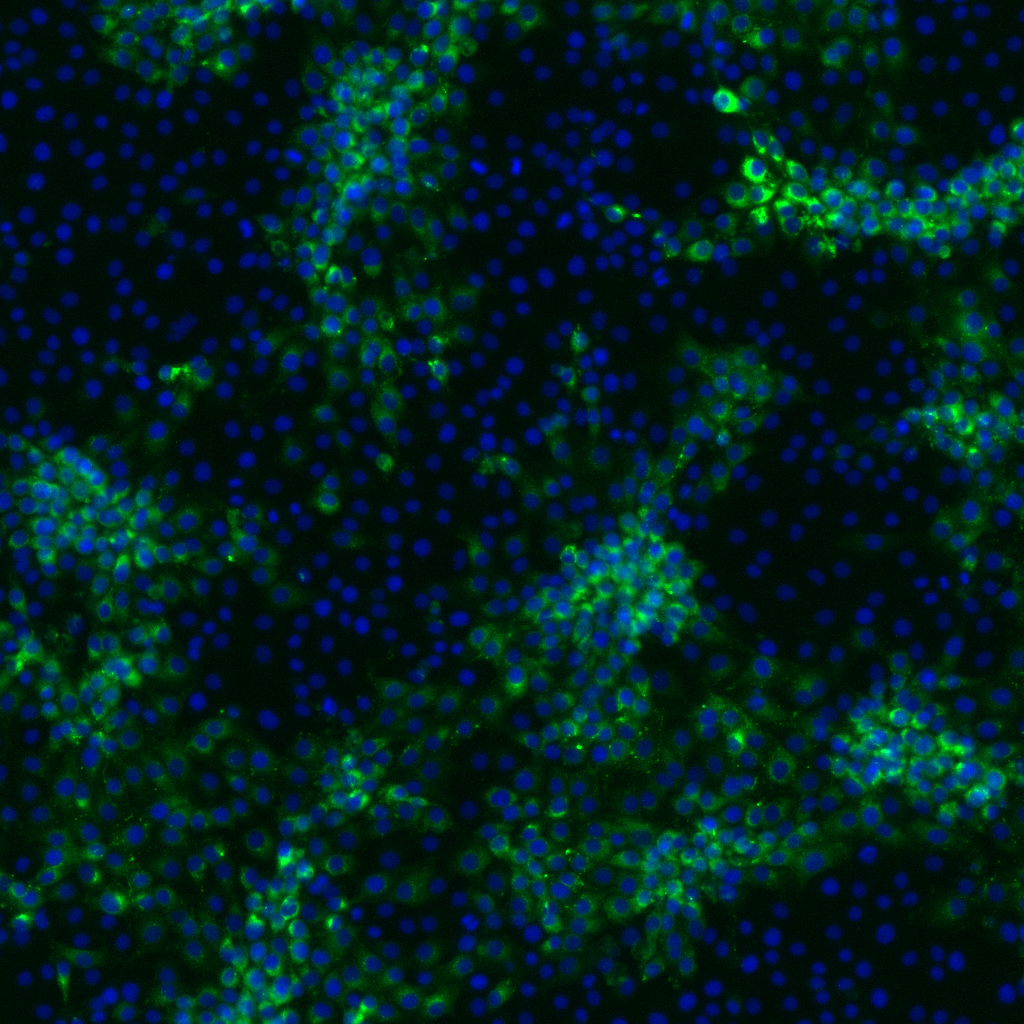

Supplement: Supplementary file 4 — Source Data [file 41467_2025_62750_MOESM4_ESM.zip › SourceDataFiles/images/Fig2g_images/prM-TBEV_pH5-5_furin+_.png]

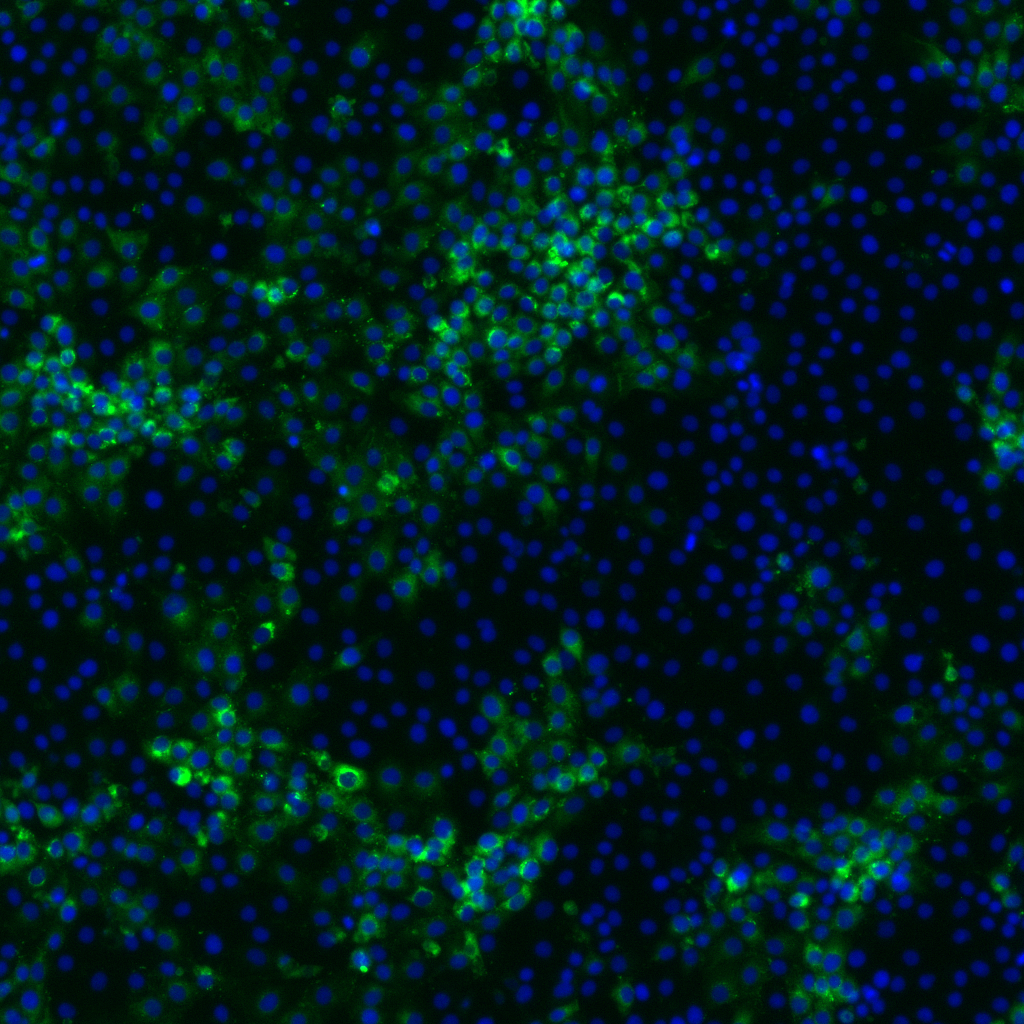

Supplement: Supplementary file 4 — Source Data [file 41467_2025_62750_MOESM4_ESM.zip › SourceDataFiles/images/Fig2g_images/prM-TBEV_pH5-5_furin-_.png]

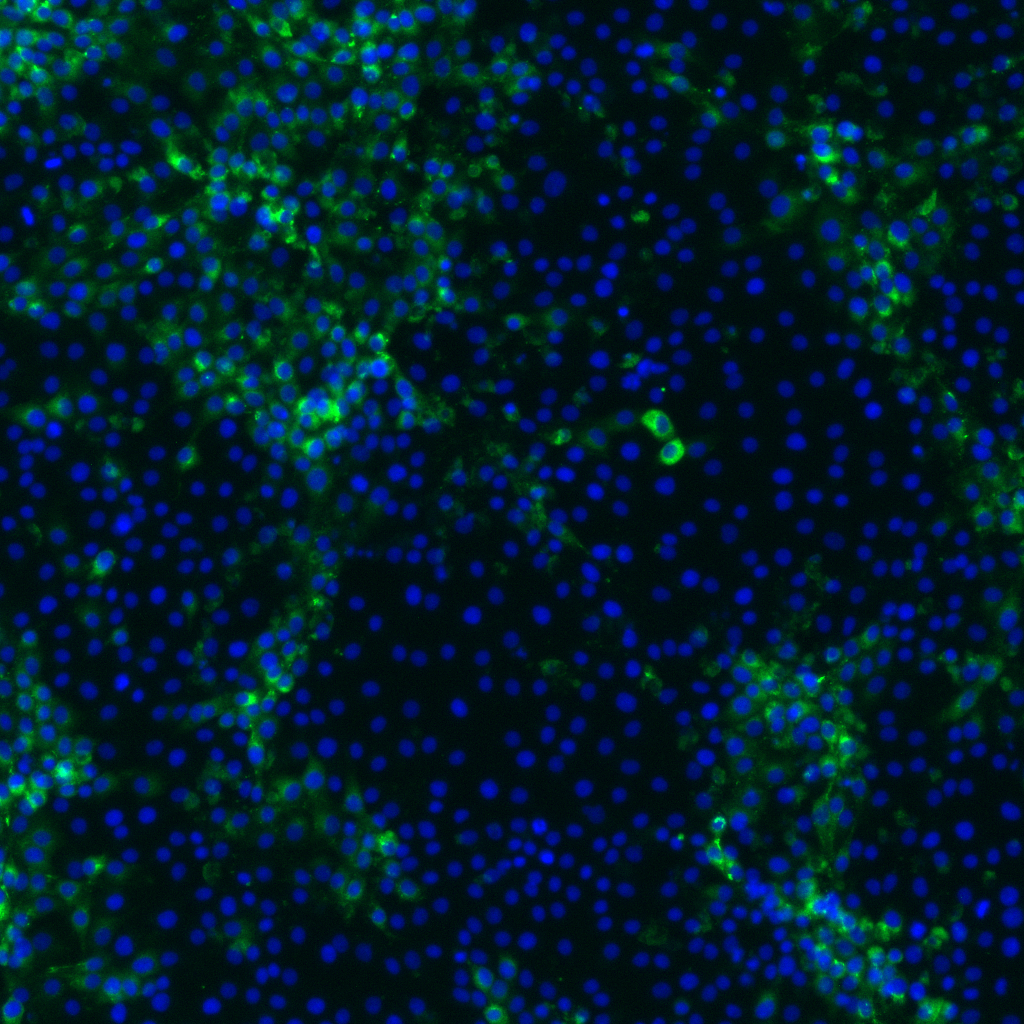

Supplement: Supplementary file 4 — Source Data [file 41467_2025_62750_MOESM4_ESM.zip › SourceDataFiles/images/Fig2g_images/prM-TBEV_pH7-5_furin+_.png]

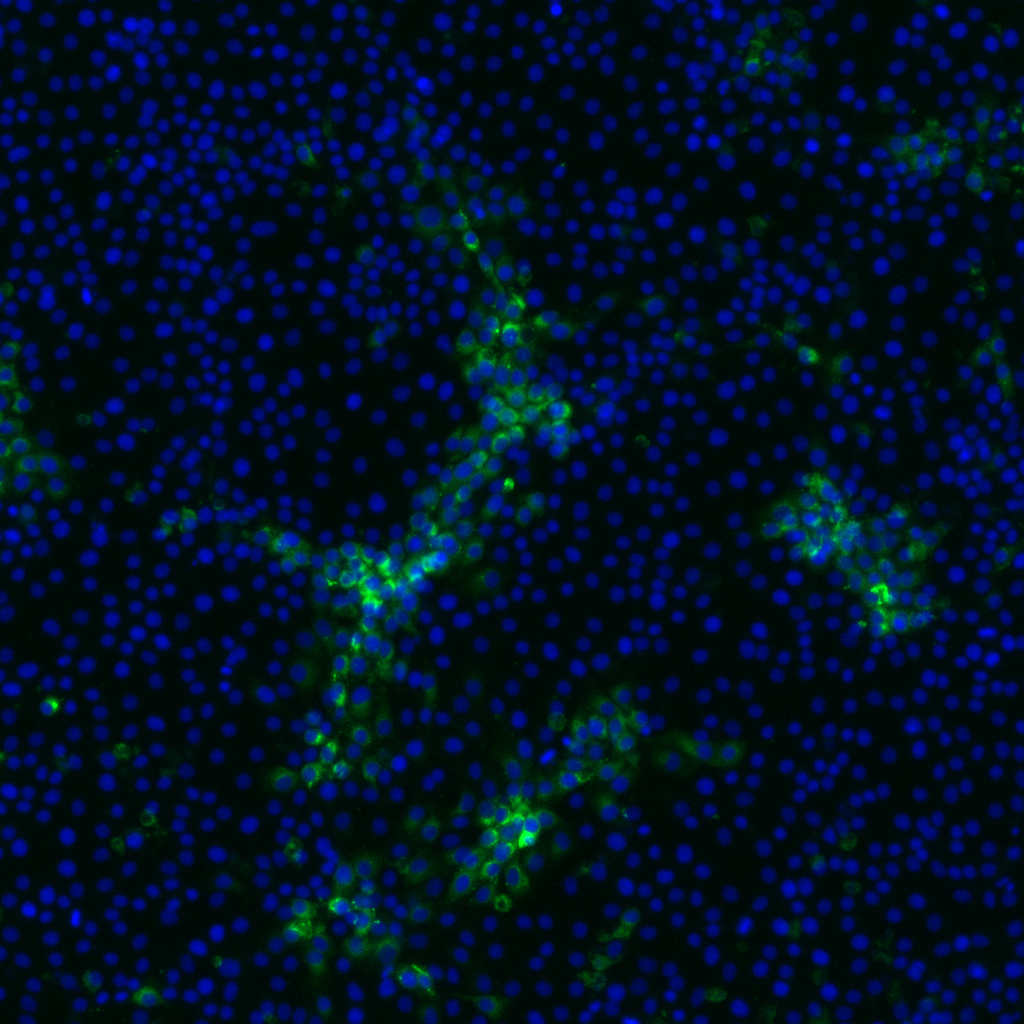

Supplement: Supplementary file 4 — Source Data [file 41467_2025_62750_MOESM4_ESM.zip › SourceDataFiles/images/Fig2g_images/prM-TBEV_pH7-5_furin-_.png]

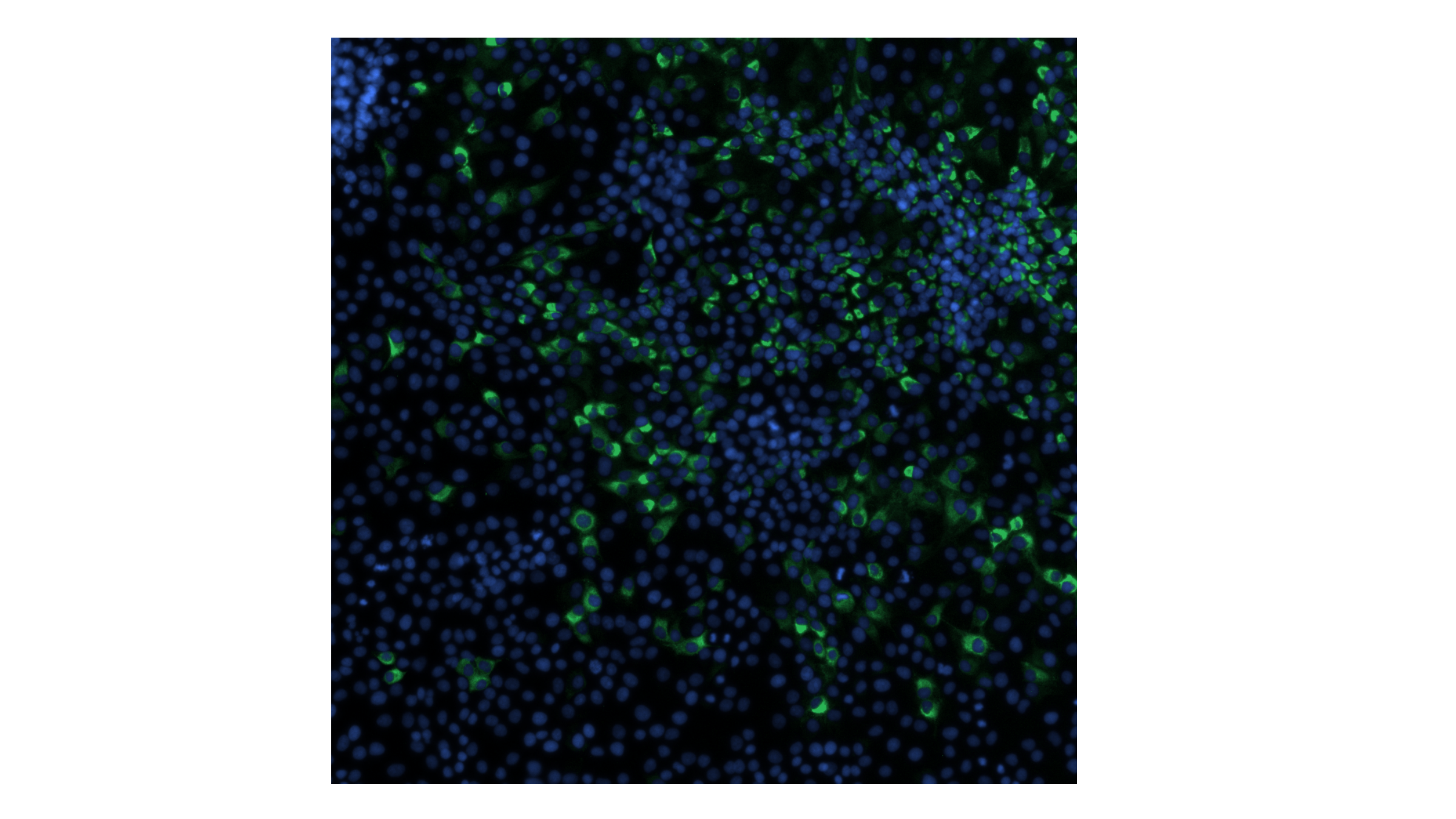

Supplement: Supplementary file 4 — Source Data [file 41467_2025_62750_MOESM4_ESM.zip › SourceDataFiles/images/Fig2g_images/usuv-2E.png]

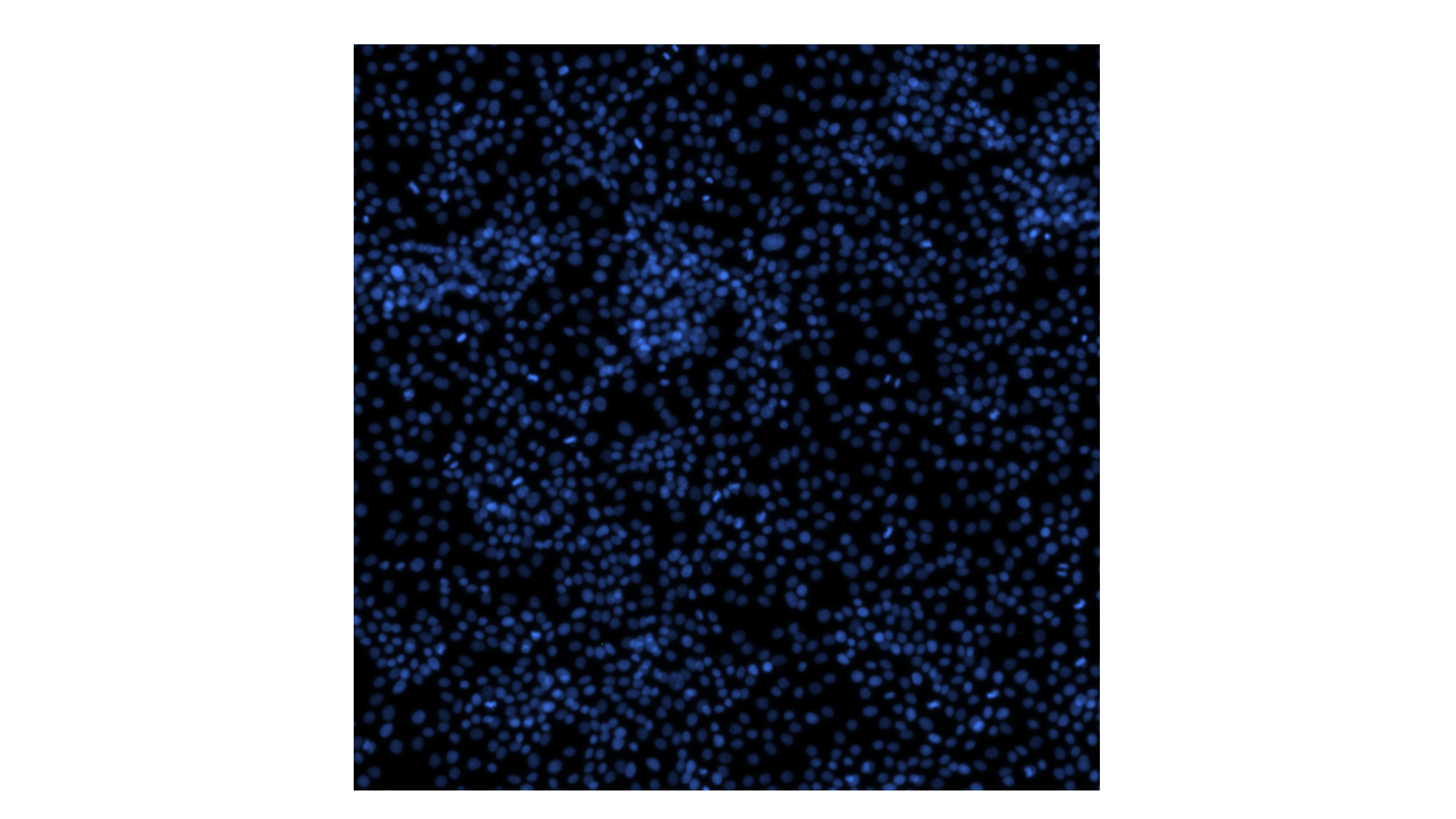

Supplement: Supplementary file 4 — Source Data [file 41467_2025_62750_MOESM4_ESM.zip › SourceDataFiles/images/Fig2g_images/usuv-B4.png]

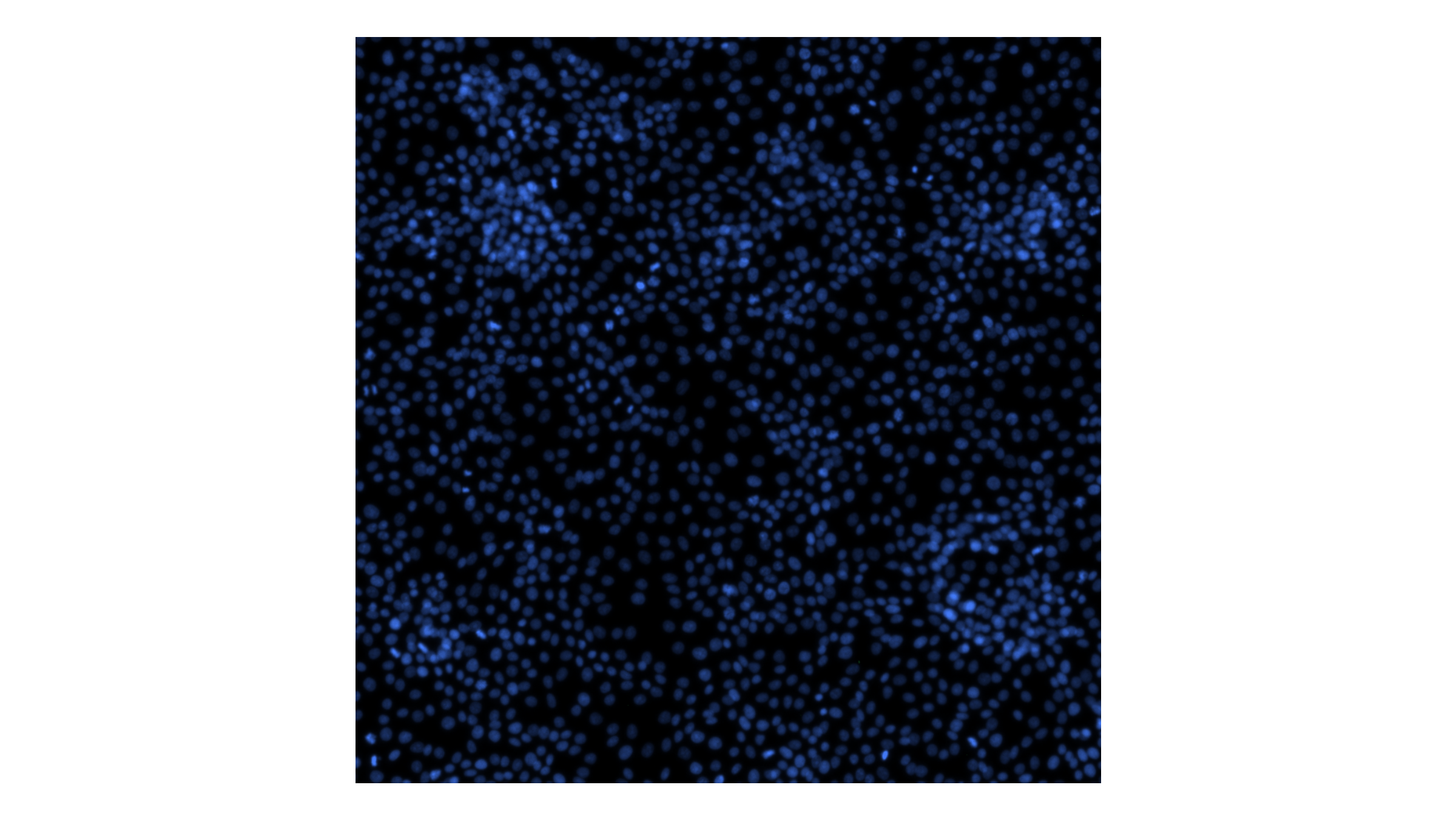

Supplement: Supplementary file 4 — Source Data [file 41467_2025_62750_MOESM4_ESM.zip › SourceDataFiles/images/Fig2g_images/usuv-C4.png]

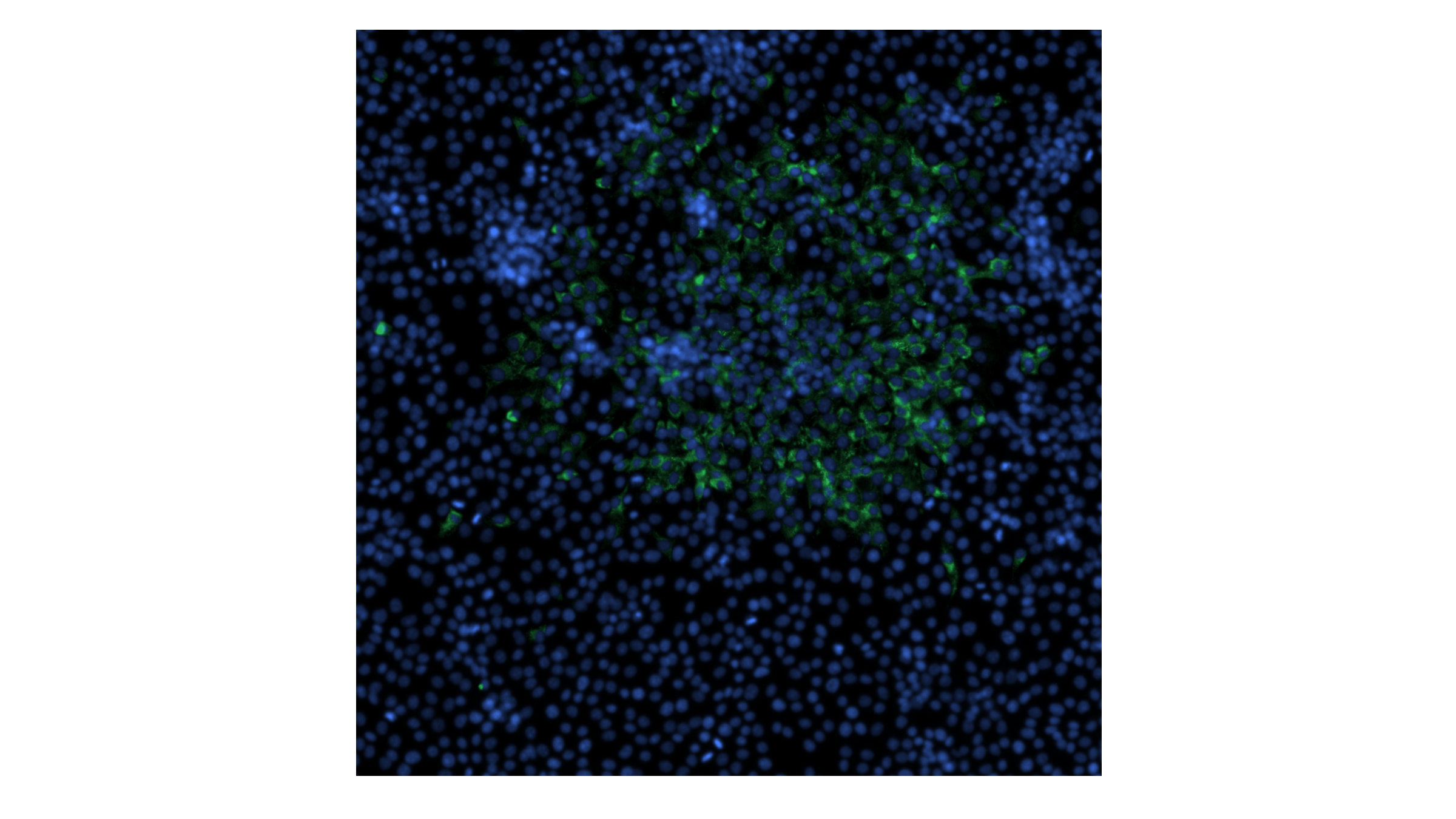

Supplement: Supplementary file 4 — Source Data [file 41467_2025_62750_MOESM4_ESM.zip › SourceDataFiles/images/Fig2g_images/usuv-E3.png]

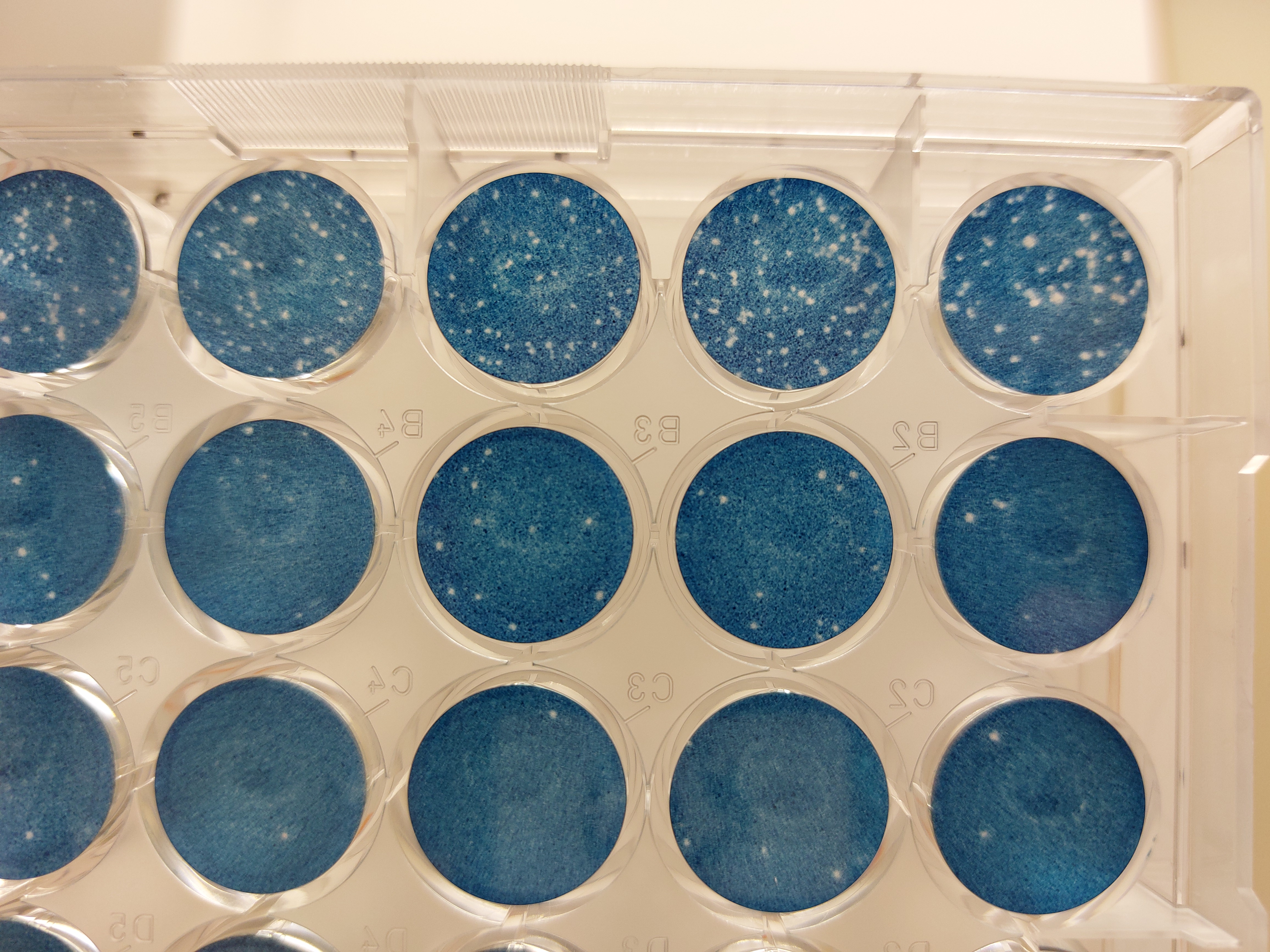

Supplement: Supplementary file 4 — Source Data [file 41467_2025_62750_MOESM4_ESM.zip › SourceDataFiles/images/m-rTBEV_plaques.jpg]

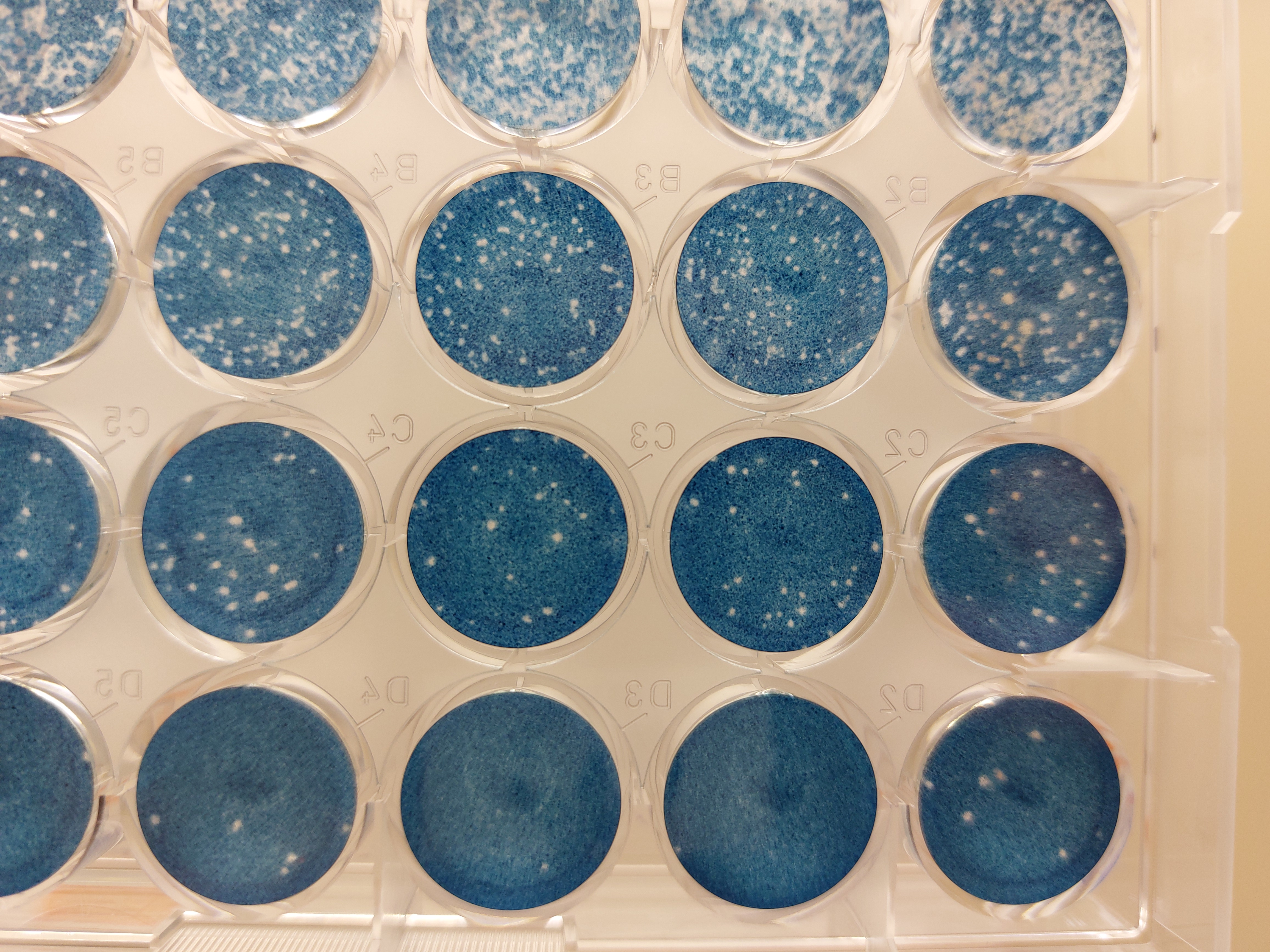

Supplement: Supplementary file 4 — Source Data [file 41467_2025_62750_MOESM4_ESM.zip › SourceDataFiles/images/m-TBEV_plaques.jpg]
